# VIRmiRNA: a comprehensive resource for experimentally validated viral miRNAs and their targets

**DOI:** 10.1093/database/bau103

**Published:** 2014-11-06

**Authors:** Abid Qureshi, Nishant Thakur, Isha Monga, Anamika Thakur, Manoj Kumar

**Affiliations:** Bioinformatics Centre, Institute of Microbial Technology, Council of Scientific and Industrial Research, Sector 39-A, Chandigarh 160036, India

## Abstract

Viral microRNAs (miRNAs) regulate gene expression of viral and/or host genes to benefit the virus. Hence, miRNAs play a key role in host–virus interactions and pathogenesis of viral diseases. Lately, miRNAs have also shown potential as important targets for the development of novel antiviral therapeutics. Although several miRNA and their target repositories are available for human and other organisms in literature, but a dedicated resource on viral miRNAs and their targets are lacking. Therefore, we have developed a comprehensive viral miRNA resource harboring information of 9133 entries in three subdatabases. This includes 1308 experimentally validated miRNA sequences with their isomiRs encoded by 44 viruses in viral miRNA ‘***VIRmiRNA***’ and 7283 of their target genes in ‘***VIRmiRtar’***. Additionally, there is information of 542 antiviral miRNAs encoded by the host against 24 viruses in antiviral miRNA ‘***AVIRmir’***. The web interface was developed using Linux-Apache-MySQL-PHP (LAMP) software bundle. User-friendly browse, search, advanced search and useful analysis tools are also provided on the web interface. VIRmiRNA is the first specialized resource of experimentally proven virus-encoded miRNAs and their associated targets. This database would enhance the understanding of viral/host gene regulation and may also prove beneficial in the development of antiviral therapeutics.

**Database URL**: http://crdd.osdd.net/servers/virmirna

## Introduction

RNA interference (RNAi) is a process in which short double stranded RNA inhibits gene expression of complementary target sequence. After the discovery of RNAi in 1998 by Fire and Mello (1), the field has gained momentum owing to various molecules being reported to regulate gene expression like microRNA (miRNA), small interfering RNA, Piwi-interacting RNA, long noncoding RNA etc. (2, 3). miRNAs are short double stranded 19–23 nucleotides long antisense molecules, which are endogenously produced and processed (4, 5). They function as regulators of gene expression by exhibiting perfect or nearly perfect base pairing with target mRNA, thus inhibiting its expression at post-transcriptional level by either mRNA degradation or translational repression (6, 7). Lin-4 was the first miRNA discovered in *Caenorhabditis* *elegans* to regulate the expression of *lin-14* protein coding gene (8). Subsequent research indicated that more than 30% of human protein-coding genes are regulated by miRNAs (9).

miRNAs play important roles in various biological processes like development, differentiation, growth, expression, cell division, stress conditions, apoptosis, cancer etc. (10, 11). They have been found to play an important role in viral infections and activation of innate immune response during disease progression (12). An increasing number of miRNAs encoded by viruses have been identified in the past. In 2004, it was found that Epstein–Barr virus (EBV) genome encodes many viral miRNAs for host/viral gene regulation (13), thereafter a number of viral miRNAs and their isomiRs were described in other viruses (14–16). IsomiRs are miRNA sequence variants that differ in length or nucleotides at the 5′ and 3′ miRNA termini and are generated from a miRNA precursor during maturation process (17). It has been reported that these isomiRs are functionally active and have evolutionary implications (18, 19).

Subsequently, host genes targeted by viral miRNAs have also been reported (20, 21). Thus, viral miRNAs help the virus to make a propagating environment in the host cell (16). Conversely, there are also reports of some antiviral miRNAs encoded by the host cell that inhibit viral pathogenesis as a means of defense mechanism (22, 23).

As miRNAs are important regulatory molecules, they may be helpful in developing novel therapeutics (24, 25). Recently, synthetic antimiRNA oligonucleotides (AMOs) have been reported to inhibit miRNAs (26). Similarly, targeting miR-122 has been reported as an antiviral strategy against hepatitis C virus (HCV) (27). miRNAs are also being exploited in other important areas like cardiac remodeling (28), inflammatory disease (29), fibrosis (30), neoangiogenesis (31) and various metabolic diseases (32). Miravirsen is the first miRNA-targeted drug to enter in the Phase IIa clinical trial with antiviral activity against HCV (24). Therefore, it would be important to have resources on viral miRNA that can prove helpful in developing antiviral therapeutics.

There are many repositories for experimental and predicted miRNAs; their cognate targets and associated interactions like miRBase (33), Plant miRNA database (PMRD) (34), microRNA.org (35), TarBase (36), miRTarBase (37), mirDB (38), miRWalk (39), miRecords (40) etc., which encompass detailed information about the miRNAs found in different organisms. There are some repositories of predicted viral miRNAs and their targets like VirMirDb (41), vhotdb (42), miRNEST (43), ViTa (44) and RepTar (45) but a dedicated experimental viral miRNA archive along with their targets is lacking. Although miRBase comprehensively covers miRNAs from diverse organisms but with limited viral miRNA sequences. Likewise, miRTarBase (37) holds knowledge of miRNA targets from different organisms, but it has very few viral miRNA targets. Therefore, a dedicated experimental viral miRNA archive along with their targets is lacking. In this study, we have developed a specialized web resource covering information on various aspects of viral miRNAs, i.e. experimentally validated miRNAs, their associated targets and antiviral miRNAs.

## Material and methods

### Data search

Literature search was performed extensively to fish out articles related to viral miRNAs, viral isomiRs (miRNA variants), targets of viral miRNAs and antiviral miRNAs from PubMed/Patent Lens. We have used following query in advanced search option of PubMed:

(((((virus) OR viruses) OR virus*) OR viral)) AND ((((((microrna) OR microrna*) OR mirna) OR mirna*) OR isomir) OR isomir*)

This query retrieved 3472 articles as on July 2014 that contains 703 review papers. We have screened these articles to shortlist over 600 potential articles after removing reviews. Articles having information pertaining to predicted miRNAs, modified nucleotides, general mechanism/expression studies, structural studies etc. were not considered. Finally data was extracted from 204 research articles having the required information.

### Database organization

For lucid presentation, viral miRNA (VIRmiRNA) resource is organized into three different categories, namely VIRmiRNA, VIRmiRtar and AVIRmir to furnish experimentally validated information of viral miRNAs, their targets and antiviral miRNAs respectively. Broadly each module provides information regarding the following fields: (i) miRNA ID, (ii) virus, (iii) taxonomy, (iv) nomenclature, (v) miRNA sequence, (vi) length, (vii) GC content, (viii) pre-miRNA, (ix) arm, (x) cell line, (xi) experimental method and (xii) references.

Additionally in VIRmiRNA sub database, outputs of the basic local alignment search tool (BLAST)/Alignment of the seed and complete miRNA sequence with that of viral as well as cellular miRNAs are provided. Predicted secondary structure of viral miRNA is also incorporated in individual entry. In VIRmiRtar and AVIRmiR sub databases, important fields like target gene name, miRNA-binding region, i.e. untranslated region or coding sequences and seed position are also given wherever available.

The database is hyperlinked to other useful resources like UniProt, GO, GenBank, PubMed and Taxonomy browser. Also, other important miRNA related resources on the web are listed separately on the ‘Related Links’ page.

### Implementation

**Figure 1.**
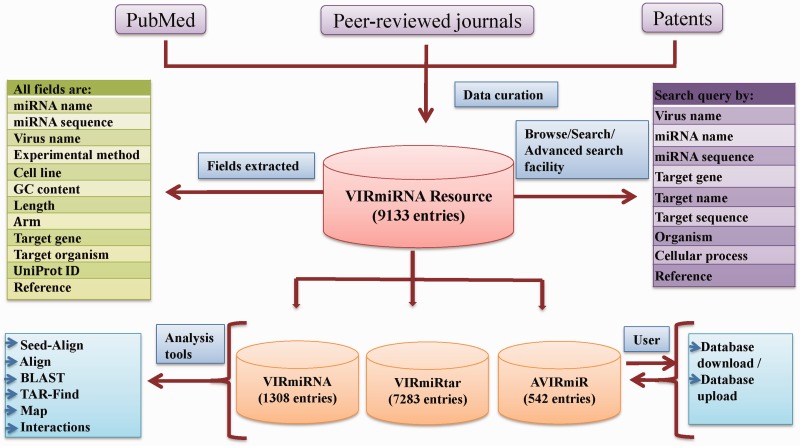
VIRmiRNA architecture.

VIRmiRNA is organized to provide easy and user-friendly access to the data. It is hosted on Red Hat Linux machine using Apache web server. The web server is maintained in MySQL at back end, while front end is implemented with PHP language. Schematic representation of VIRmiRNA architecture is shown in Figure 1.

## Results

### Database statistics

**Figure 2.**
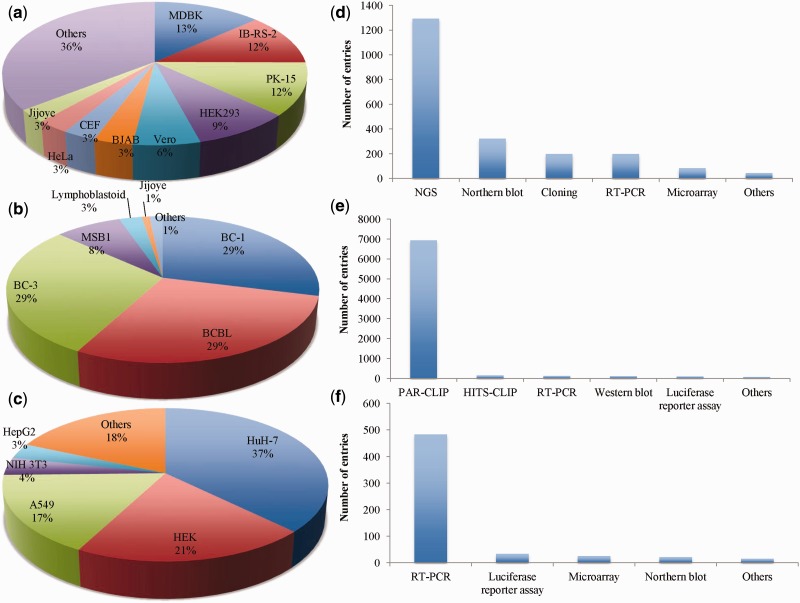
VIRmiRNA statistics: Pie charts (**a–c**) representing statistical distribution of cell lines and bar graphs (**d–f**) depicting the statistical trend of experimental methods used in the three subdatabases viz VIRmiRNA, VIRmiRtar and AVIRmiR, respectively. NGS, next generation sequencing; RT-PCR, real-time PCR; CLIP, cross-linking immunoprecipitation.

VIRmiRNA resource encompasses experimentally validated information of 9133 entries divided into three subarchives, namely VIRmiRNA, VIRmiRtar and AVIRmiR. Detailed statistics of each of the module is depicted in Figure 2.

VIRmiRNA subdatabase has information of 1308 viral miRNA including 538 isomiRs sequences that belong to 44 viruses infecting a range of host organisms, namely human (17 viruses), other mammals (16 viruses), birds (7 viruses), invertebrates (3 viruses) and plants (1 virus). It includes a maximum of 287 miRNAs of Pseudorabies virus (PRV) followed by 111 miRNAs of Rhesus lymphocryptovirus (RLCV) and miRNAs of other important viruses like EBV, Herpes simplex virus (HSV) and Duck enteritis virus (DEV) etc. (Supplementary Table S1). These miRNAs have been validated on different cell lines like IB-RS-2, PK-15, HEK, BC, chicken embryo fibroblasts (CEF), Vero cells etc. (Figure 2a). Experimental methods used were Northern blot, real-time polymerase chain reaction (RT-PCR), microarray analysis, cloning and next generation sequencing (NGS) (Figure 2d), out of which NGS was the most commonly used experimental platform to identify viral miRNAs.

VIRmiRtar harbors 7283 experimentally validated viral miRNA targets from 15 different viruses like EBV, Kaposi sarcoma-associated herpesvirus (KSHV), Human cytomegalovirus (HCMV) and HSV-1 etc. Maximum 3683 and 2126 miRNA targets have been reported for EBV and KSHV, respectively (Supplementary Table S2). Targets have been identified using different cell-lines like BC-1, body-cavity-based lymphoma cell line (BCBL), BC-3, Lymphoblastoid, MSB1, Jijoye and others (Figure 2b). These viral miRNA targets have been determined using different experimental methods like photoactivatable ribonucleoside-enhanced crosslinking and immunoprecipitation (21, 46), high-throughput sequencing and cross-linking immunoprecipitation (47, 48), RT-PCR (48), western blot (49), luciferase reporter assay (50), etc. (Figure 2e).

It has been reported earlier that some genes were being targeted by more than one miRNA (51, 52). Similar trend was observed for viral miRNA targets like CCNT2, BRWD1, BTBD3, FNDC3A, LCOR etc. that were under the regulation of multiple miRNAs as briefed in Table 1 and detailed in Supplementary Table S4.

**Table 1.** Host genes targeted by multiple viral miRNAs

S. No.	Target	Viral miRNAs	No. of miRNA	Description	UniProt ID	Reference
1	CCNT2	kshv-miR-k12-7, ebv-miR-bart9, ebv-miR-bart1-3p, ebv-miR-bart10, kshv-miR-k12-5, kshv-miR-k12-1	6	Cyclin-T2	F2Z2C9	22100165, 22291592, 22473208
2	BRWD1	kshv-miR-k12-11, kshv-miR-k12-6-3p, ebv-miR-bart6-3p, ebv-miR-bart4, ebv-miR-bart3	5	Bromodomain and WD repeat-containing protein 1	Q9NSI6	22100165, 22291592
3	BTBD3	ebv-miR-bart8*, kshv-miR-k12-9, ebv-miR-bart10, kshv-miR-k12-5, ebv-miR-bart15	5	BTB/POZ domain-containing protein 3	F8WAQ4	22100165
4	FNDC3A	kshv-miR-k12-5, ebv-miR-bart6-3p, ebv-miR-bart2-5p, ebv-miR-bart14, ebv-miR-bart3	5	Fibronectin type III domain containing 3A, isoform CRA_f	G5E9X3	22100165, 22291592
5	LCOR	kshv-miR-k12-10b, kshv-miR-k12-4-3p, ebv-miR-bart9*, ebv-miR-bart19-3p, ebv-miR-bart4	5	Ligand-dependent corepressor	Q96JN0	22100165, 22291592
6	LMBR1	kshv-miR-k12-9*, ebv-miR-bart20-3p, kshv-miR-k12-6-3p, ebv-miR-bart3*, ebv-miR-bart15	5	Limb region 1 protein homolog	F8WDW0	22100165, 22291592
7	MEQ	mdv1-miR-m1, mdv1-miR-m2-5p, mdv1-miR-m3, mdv1-miR-m4, mdv1-miR-m5	5	Oncoprotein MEQ	Q9DGW5	16912324
8	MLL	kshv-miR-k12-9, ebv-miR-bart6-5p, ebv-miR-bart10, kshv-miR-k12-1, ebv-miR-bart19-3p	5	Histone-lysine *N*-methyltransferase 2A	Q03164	22100165, 22473208
9	NCOR1	kshv-miR-k12-12, ebv-miR-bart8, ebv-miR-bart20-3p, kshv-miR-k12-1, ebv-miR-bart16	5	Nuclear receptor corepressor 1	O75376	22100165
10	PRPF40A	kshv-miR-k12-10a, ebv-miR-bart19-3p, kshv-miR-k12-2, ebv-miR-bart14, ebv-miR-bhrf1-2	5	Pre-mRNA-processing factor 40 homolog A	H7BXZ7	22100165, 22291592

The third submodule, i.e. AVIRmiR deals with small yet quite important data about antiviral miRNAs. It contains 542 antiviral miRNAs acting against 26 different viruses (Supplementary Table S3). These miRNAs were predominantly tested using HuH-7, HEK, A549, NIH-3T3 and HepG2 cell lines (Figure 2c). These miRNAs have been mostly validated using RT-PCR, luciferase reporter assay, microarray and northern blot (Figure 2f). It has been described earlier that a miRNA can target numerous genes (51). We also found hsa-miR-181b-5p, hsa-miR-142-5p, hsa-let-7 regulate many genes as shown in Table 2 and Supplementary Table S5.

**Table 2.** Antiviral miRNAs targeting multiple viral genes

S. No.	miRNA name	No. of targets	Target name	Virus name	Biological process involved	Reference
1	hsa-miR-181b-5p	10	CD163, M1, M2, NA, NEP, NP, NS1, PA, PB1, PB2	INFV, VZV, PRRSV	Replication	WO/2010/101663/A2, 23740977, 22676898, 20643945,20643939
2	hsa-miR-142-5p	10	M1, M2, NA, NEP, NP, NS1, PA, PB1, PB2, 3' UTR	INFV, DENV	Replication	22241991, WO/2010/101663/A2
3	hsa-let-7	9	Casp3, DICER1, HMGA2, IFN-beta, IGF2BP1, IGF2BP2, MYC, RAB40C, STAT3	HBV, VZV, HCV	Replication, translation, interferon system	21565290, 18668040, 23824794, 20969775, 18700235, 20643939
4	hsa-miR-149	9	M1, M2, NEP, NP, NS1, PA, PB1, PB2, vpr	HIV, INFV	Replication, translation	16236258, WO/2010/101663/A2
5	hsa-miR-16	9	M1, M2, Nef, NEP, NP, NS1, PA, PB1, PB2	INFV, HIV	Replication	22080513, WO/2010/101663/A2
6	hsa-miR-17-5p	9	M1, M2, Nef, NEP, NP, NS1, PA, PB1, PB2	INFV, HIV	Replication	17322031, WO/2010/101663/A2
7	hsa-miR-93	9	IRES, M1, M2, NEP, NP, NS1, PA, PB1, PB2	INFV, VSV	Replication	21431677, WO/2010/101663/A2
8	hsa-miR-122	8	5' UTR, CAT-1, CCNG1, HBsAg, HO-1, IFN-beta, NDRG3, SOX6	VZV, BDV, HCV	Replication, translation	20561966, 18431360, 21725618, 21565290, 21821155
9	hsa-miR-1259	8	M1, M2, NEP, NP, NS1, PA, PB1, PB2	INFV	Replication, interferon system	22676898, 20643939, 18431360, 20561966, 21565290, 21821155, 16141076
10	hsa-miR-1977	8	M1, M2, NEP, NP, NS1, PB1, PB2, PA	INFV	Replication	WO/2010/101663/A2

### Web server interface

We have provided search, advanced search and browse options to retrieve the desired data from each of the three different subdatabases.

#### Database search

**Figure 3.**
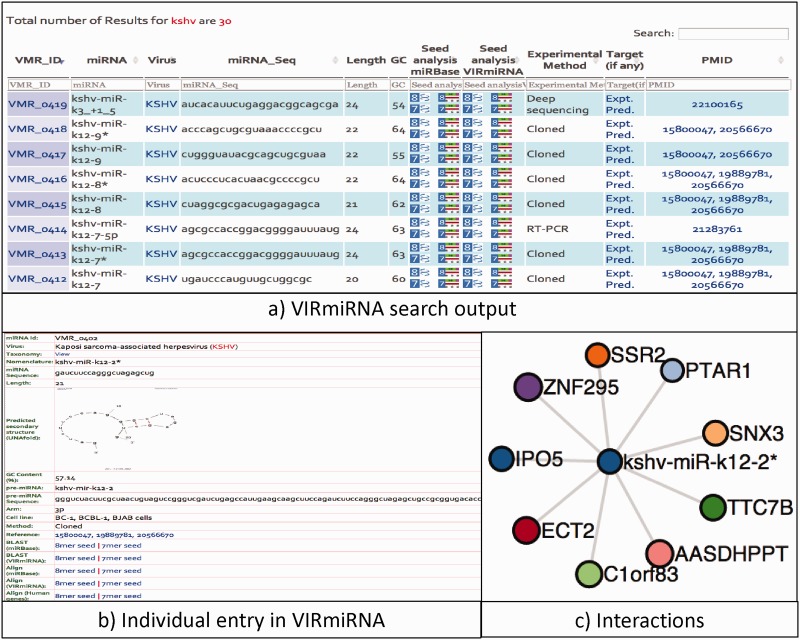
Schematic representation of the search output (**a**) a screenshot of search output returned in the tabular form having information regarding KSHV miRNAs, (**b**) Detailed information of kshv-miR-k12-2* miRNA, (**c**) and its interaction partners in graphical view.

In the search menu option, user can select any of the three submodules to search for a given keyword in all or specific fields. The search output provides information on the various aspects of miRNAs, i.e. nomenclature, sequence, length, GC content, cell line, experimental method and reference. Additionally, internal links showing the output of tools like Seed BLAST/Align (8mer/7mer) of miRNA seed sequence with that of viral miRNA (VIRmiRNA) and cellular ones (miRBase) is displayed. In search output, we have also provided sorting and filtering functionality. Also, we have made the search results interactive by interlinking a viral miRNA entry with its experimental/predicted targets. By clicking on the miRNA ID, user can get complete details of that record. Result of the search output for KSHV miRNA is shown in the Figure 3.

#### Advanced search

Advanced search using logical operators helps the user to find the appropriate data stringently in each of the subdatabases. User can successively build the query by entering the desired keyword and selecting the corresponding field and logical operators (AND/OR). For example in *VIRmiRNA*, on searching EBV in the ‘Virus’ field, the output displays 52 entries, then by selecting ‘Method’ field as Northern blot, the output shows 46 entries. Further by choosing ‘Length’ field as 22, the final result output lists 26 entries.

#### Database browsing

*VIRmiRNA* browse gives a list of viruses along with the number of miRNAs for that virus, e.g. HSV-1 has 48 miRNAs. On clicking a virus name, information of all the miRNAs encoded by that virus will be displayed, and further details can be viewed by clicking on its ID. In order to know the classification of the virus, we have also provided an external link to ‘NCBI Taxonomy browser’ for each virus. *VIRmiRtar* browse provides a list of viral miRNAs along with the number of their target genes. For example, ebv-miR-bart19-3p has maximum of 370 targets, which can be viewed by clicking on the particular miRNA. *AVIRmiR* browse displays a list of viruses along with the number of antiviral miRNAs and virus taxonomy.

### Analysis tools

We have also integrated various tools in VIRmiRNA like Seed-Align, BLAST, Map and TarFind for the analysis of the miRNA sequences. In *VIRmiRNA-Seed-Align*, user can align any given miRNA seed sequence against VIRmiRNA as well as cellular (miRBase) miRNAs. User has to provide the miRNA seed sequence in FASTA format. The output provides a list of matching seed sequences along with their database accession numbers, nomenclature, alignment positions and percent identity values.

*VIRmiRNA-BLAST* searches the presence of miRNA sequences in the database matching the user provided miRNA sequence. The output shows both graphical and text alignment of the matching miRNA sequences along with their alignment score. *VIRmiRNA-Map* displays the perfectly matching miRNAs available in our database against a given nucleotide sequence. It helps user to find how many miRNAs are available in the database corresponding to the user provided sequence. *VIRmiRNA-TarFind* predicts the target genes in the human reference gene sequence against the user provided miRNA or seed sequence. *VIRmiRNA-Update* helps user to submit new viral miRNA information, which will be included in the main database after cross checking. We have also included ‘How to use’ and Frequently Asked Questions (FAQ) pages as well as ‘help pop-ups’ on individual pages to further assist the user to effectively use this resource. The ‘How to use’ page is given with snapshots of input and output examples. Besides, user can download any of the three subdatabases from the ‘Downloads’ section.

## Discussion

miRNAs are considered as important noncoding RNA molecules playing a fundamental role in the regulation of cellular pathways (53, 54) and viral infection (16, 55). There are various experimentally validated miRNA databases reported in the literature like miRBase—a general miRNA storehouse (56), PMRD—a plant miRNA repository (34), miRCancer—a cancer-associated miRNA assemblage (57) etc. Of these only miRBase (56), the largest primary miRNA sequence repository, contains limited information on viral miRNAs. In VIRmiRNA, we have provided updated and comprehensive information on viral miRNAs from as many as 42 different viruses, which is 2-fold more than any existing resource. In addition, many resources of predicted miRNAs like miRGen (58), miRWalk (39), mirDB (38), microRNA.org (35) also exist. However, for viral miRNA only a few predicted databases have been described like VirMirDb—a predicted viral miRNA database (41), vhotdb—a collection of predicted host–virus miRNA interactions (42) and miRNEST (43) but they do not furnish information on experimental viral miRNAs.

It has been reported that a single miRNA gene can express multiple isomiRs which differ between cell and tissue types (18). The isomiRs may either form an miRNA locus to coordinate target mRNA regulation (19) or their target transcripts could also potentially differ (17). VIRmiRNA also harbors knowledge about viral isomiRs that would be helpful in miRNA analysis. Studies have shown that some viral miRNAs have their orthologous cellular counterparts due to identical or partial overlap seed region. For e.g. kshv-miR-K12-11 has been reported to be orthologous to cellular miR-155 (59, 60), kshv-miR-K10a to miR-142-3p (21) and BLV-miR-B4 to hsa-miR-29 (61). For the analysis of miRNA mimics, we have integrated seed align tool, which will be helpful to find miRNAs with identical seed region in both cellular and viral miRNAs.

There are a few repositories of experimentally validated miRNA targets viz. TarBase (36), miRNAMap 2.0 (62) and miRTarBase (37). Although miRTarBase has comprehensive information of miRNA targets from several organisms, but it has scarce data on viral miRNA targets. Simultaneously, there are many catalogues of predicted miRNA targets viz. TargetScan (63), PicTar (64), mirDB (38), DIANA microT (65), miRanda (51), ComiR (66). Moreover, databases of predicted viral miRNA targets are also available including ViTa—a database of predicted host miRNA targets on the viral genome (44) and RepTar—a repository of predicted cellular targets of mammalian as well as viral miRNAs (45). Comparatively, VIRmiRtar is the only database that holds updated and inclusive information of over 7000 experimental viral miRNA targets from 15 viruses.

It has been reported that multiple miRNAs bind to a single target gene, a phenomenon known as cooperativity (51, 52). Analysis of the target genes in VIRmiRtar showed that CCNT2, BRWD1, BTBD3, FNDC3A and LCOR etc. genes were targeted by several miRNAs. CCNT2 or Cyclin-T2 that functions as a regulator of CDK9 kinase (67) is being targeted by six miRNAs, belonging to KSHV and EBV, namely kshv-miR-k12-7, ebv-miR-bart9, ebv-miR-bart1-3p, ebv-miR-bart10, kshv-miR-k12-5 and kshv-miR-k12-1. Similarly, BRWD1 or Bromodomain and WD repeat-containing protein-1, that controls cell shape via the regulation of cytoskeletal organization (68) is being regulated by five viral miRNAs. Yet another gene BTBD3 or BTB/POZ domain-containing protein 3, that functions as a regulator of mammalian neurodevelopmental processes (69) is also being targeted by five different viral miRNAs. These and other important viral miRNA targets are briefly listed in Table 2.

Using KEGG Mapper, we found that the target genes in VIRmiRtar were enriched in several important cellular pathways including Metabolic (hsa01100), cancer (hsa05200), viral carcinogenesis (hsa05203) pathways with 188, 96 and 84 hits, respectively. Other important pathways with significant hits include viral infections, MAPK signaling pathway, PI3K-Akt and cell cycle (Supplementary File S2). Thus, the viral miRNA targets include a range of genes that regulate diverse biological pathways. The role of miRNAs as key gene regulators in different cellular pathways has further been reviewed (9, 70, 71).

Scientists have studied that viral pathogenesis is regulated by host miRNAs which target viral or cellular genes necessary for virus replication (72), e.g. eight interferon-β regulated human miRNAs have been found to target HCV (73). AVIRmiR is the only repository having information of host miRNAs showing antiviral effect. Research findings have stated that a particular miRNA targets more than one gene, a phenomenon termed as ‘multiplicity’ (51). In AVIRmiR, we found the similar observation that antiviral miRNAs target multiple genes e.g. hsa-miR-181b-5p, which functions as a tumor suppressor (74), inhibits ten viral genes from different viruses like influenza A virus (INFV A), Porcine Reproductive and Respiratory Syndrome Virus (PRRSV), Varicella-zoster virus (VZV) and Mink enteritis virus. Similarly, hsa-miR-142-5p targets nine different genes of INFV and Dengue virus (DENV), while hsa-miR-16 and hsa-miR-17-5p are reported to target nine different genes of human immunodeficiency virus (HIV) and INFV (Table 2). It appears from the trend of multiple gene regulation by antiviral miRNA that they are adapted to target genes critical for virus survival and thus play a vital role in host antiviral defense.

## Conclusion

Understanding different aspects of viral miRNA biology may be harnessed in combating viral infections. In VIRmiRNA resources, we have furnished updated and exhaustive information on experimentally validated viral encoded miRNAs with their isomiRs, the largest collection of their associated targets and the first catalogue of antiviral miRNAs. This comprehensive resource along with the analysis tools would be useful in deciphering host–virus interactions and the development of miRNA based therapeutics against the pathogenic viruses.

## Future implications

In the future, our main focus will be to update the resource by including new viruses and targets, once the appropriate information is available in the literature.

## Supplementary Data

Supplementary data are available at *Database* Online.

Supplementary Data
