# Supplementary material for: VIRmiRNA: a comprehensive resource for experimentally validated viral miRNAs and their targets
Source: Database (Oxford). 2014 Nov 6;2014:bau103. doi: 10.1093/database/bau103 (PMC4224276; doi:10.1093/database/bau103)
Supplement: Supplementary Data [file supp_2014_bau103_index.html]

VIRmiRNA: a comprehensive resource for experimentally validated viral miRNAs and their targets — Supplementary Data 

# VIRmiRNA: a comprehensive resource for experimentally validated viral miRNAs and their targets

## Supplementary Data

files

**Files in this Data Supplement:**

- Supplementary Data - doc file
- Supplementary Data - doc file
