# Supplementary material for: VIRmiRNA: a comprehensive resource for experimentally validated viral miRNAs and their targets
Source: Database (Oxford). 2014 Nov 6;2014:bau103. doi: 10.1093/database/bau103 (PMC4224276; doi:10.1093/database/bau103)
Supplement: Supplementary Data [file supp_bau103_Supplementary_1_R2.doc]

**Supplementary Material**

**Supplementary File 1**

**Table S1:** Showing distribution of viral miRNA of different viruses in VIRmiRNA sub-database

**Table S2:** Showing distribution of viral miRNA target genes of different viruses in VIRmiRtar sub-database

**Table S3:** Showing distribution of anti-viral miRNAs of different viruses in AVIRmiRNA sub-database

**Table S4:** Host genes targeted by multiple viral miRNAs in VIRmiRtar sub-database

**Table S5:** Table depicting AVIRmiRNAs and their multiple viral genes along with their role

**Table S1**. Distribution of viral miRNA of different viruses in VIRmiRNA sub-database

| **S. No.** | **Virus name** | **No. of viral miRNAs** | **Reference** |
| --- | --- | --- | --- |
| 1 | Bovine foamy virus (BFV) | 20 | 24522910 |
| 2 | BK polyomavirus (BKV) | 2 | 18684810, 24735545 |
| 3 | Bovine leukemia virus (BLV) | 15 | 22308400, 23345446 |
| 4 | Bombyx mori nucleopolyhedrosis virus (BmNPV) | 5 | 20800868 |
| 5 | Bovine herpesvirus 1 (BoHV1) | 12 | 19793906 |
| 6 | Bovine herpesvirus 5 (BoHV5) | 5 | 24385436 |
| 7 | Bandicoot papillomatosis carcinomatosis virus type 1 (BPCV1) | 1 | 21345962 |
| 8 | Bandicoot papillomatosis carcinomatosis virus type 2 (BPCV2) | 1 | 21345962 |
| 9 | Duck enteritis virus (DEV) | 33 | 22492913 |
| 10 | Epstein Barr virus (EBV) | 52 | 15118162, 16540699, 16557291, 19091858, 19144710, 22291592, 20808852, 17604727, 19881953, 22100165 |
| 11 | Hepatitis C virus | 1 | 19144437 |
| 12 | Herpes B virus (HBV) | 74 | 19144716, 21543500 |
| 13 | Human cytomegalovirus (HCMV) | 28 | 15782219, 16140786, 17983268, 22715351, 22013051 |
| 14 | Human herpesvirus 6B (HHV-6) | 8 | 22114334 |
| 15 | Human immunodeficiency virus 1 (HIV-1) | 4 | 15601474, 15782219, 18299284, 15722536 |
| 16 | Human Papillomavirus (HPV) | 9 | 23936163 |
| 17 | Herpes simplex virus 1 (HSV1) | 48 | 16699030, 18596690, 20181707, 22661375, 23536669, 23512275, 19656888 |
| 18 | Herpes simplex virus 2 (HSV2) | 24 | 19889786, 20181707, 19019961 |
| 19 | Heliothis virescens ascovirus (HvAV) | 1 | 18614632 |
| 20 | Herpesvirus saimiri strain A11 (HVSA) | 6 | 21925386 |
| 21 | Herpesvirus of turkeys (HVT) | 28 | 19328516, 19403687 |
| 22 | Infectious laryngotracheitis virus (ILTV) | 10 | 19328516, 19728068 |
| 23 | JC polyomavirus (JCV) | 2 | 18684810 |
| 24 | Kaposi sarcoma-associated herpesvirus (KSHV) | 30 | 15782219, 15800047, 16540699, 17500590, 21283761, 19889781, 20566670, 15994824, 22100165 |
| 25 | Mouse cytomegalovirus (MCMV) | 28 | 17928340, 17942535 |
| 26 | Merkel cell polyomavirus (MCV) | 2 | 19046593 |
| 27 | Mareks disease virus type 1 (MDV1) | 35 | 16912324, 18256158, 18430245, 21645299, 18842708, 24449754 |
| 28 | Mareks disease virus type 2 (MDV2) | 40 | 17459919, 19328516, 24449754 |
| 29 | Mouse gammaherpesvirus 68 (MGHV) | 29 | 15782219, 20668074, 23110115, 20660200 |
| 30 | Ovine herpesvirus 2 (OvHV-2) | 35 | 21957125, 24849241 |
| 31 | Pseudorabies virus (PRV) | 287 | 22292087 |
| 32 | Murine Polyomavirus (PyV) | 1 | 19272626 |
| 33 | Rat cytomegalovirus (RCMV) | 60 | 20980502 |
| 34 | Rhesus Cytomegalovirus (RhCMV) | 17 | 22305624 |
| 35 | Rhesus lymphocryptovirus (RLCV) | 111 | 16557291, 19889779, 20219930 |
| 36 | Rhesus rhadinovirus (RRV) | 97 | 17451774, 20655562 |
| 37 | Simian Agent Polyomavirus (SA12) | 1 | 16189011 |
| 38 | Semliki Forest virus (SFV) | 1 | 23077310 |
| 39 | Singapore grouper iridovirus (SGIV) | 16 | 21559453 |
| 40 | Simian virus 40 (SV40) | 2 | 15931223 |
| 41 | Torque teno virus (TTV) | 4 | 24367263 |
| 42 | Turnip mosaic virus (TuMV) | 82 | WO 2012/048385 A1 |
| 43 | West Nile Virus (WNV) | 1 | 22080551 |
| 44 | White Spot Syndrome virus (WSSV) | 40 | 22832246 |

**Table S2**. Distribution of viral miRNA target genes of different viruses in VIRmiRtar sub-database

| **S. No.** | **Virus name** | **No. of Targets** | **Reference** |
| --- | --- | --- | --- |
| 1 | Bombyx mori nucleopolyhedrosis virus (BmNPV) | 1 | 22593162 |
| 2 | Bovine leukemia virus (BLV) | 2 | 22308400 |
| 3 | Epstein Barr virus (EBV) | 3683 | 22291592, 22473208, 18316607, 19881953, 18838543, 18073197, 17911266, 19380116, 24586173, 24899173, 20716523, 20413099, 21379335, 22291590, 22100165, 25012295, 20862214 |
| 4 | Herpes simplex virus 1 (HSV1) | 7 | 23536669, 22787211, 18596690, 23512275 |
| 5 | Herpes simplex virus 2 (HSV2) | 1 | 22787211 |
| 6 | Human cytomegalovirus (HCMV) | 33 | 23403649, 21900172, 21690488, 21219658, 19380116, 20585629, 17983268, 19656885, 22645331, 23938381, 17641203, 17983260 |
| 7 | Human immunodeficiency virus 1 (HIV-1) | 3 | 22558366, 19082544 |
| 8 | Infectious laryngotracheitis virus (ILTV) | 1 | 21232778 |
| 9 | Kaposi sarcoma-associated herpesvirus (KSHV) | 2126 | 23185331, 18075594, 19098914, 20844036, 22896623, 22915806, 22174674, 22100165, 21813606, 21616184, 21402938, 20219912, 20080955, 20081837, 20071580, 17500590, 20413099, 19380116, 18378902, 17881434, 21283761, 20006845, 21616180 |
| 10 | Mareks disease virus type 1 (MDV1) | 560 | 20962090, 16912324, 24449754 |
| 11 | Mareks disease virus type 2 (MDV2) | 859 | 24449754 |
| 12 | Semliki Forest virus (SFV) | 2 | 23077310 |
| 13 | Simian virus 40 (SV40) | 3 | 22558366 |
| 14 | Torque teno virus (TTV) | 1 | 24367263 |
| 15 | West Nile Virus (WNV) | 1 | 22080551 |

**Table S3**. Distribution of antiviral miRNAs of different viruses in AVIRmiRNA sub-database

| **S. No.** | **Virus** | **No. of antiviral miRNAs** | **Reference** |
| --- | --- | --- | --- |
| 1 | Avian leukosis virus (ALV) | 5 | 22188662, 23907393 |
| 2 | Borna disease virus (BDV) | 1 | 20561966 |
| 3 | Coxsackievirus B type (CVB3) | 1 | 22197249 |
| 4 | Dengue virus (DENV) | 1 | 22241991 |
| 5 | Enterovirus (EV71) | 3 | 23594713, 23468506 |
| 6 | Epstein Barr Virus (EBV) | 3 | 18039120, 12007417 |
| 7 | Foot-and-mouth disease virus (FMDV) | 4 | 21663611 |
| 8 | Hepatitis B virus (HBV) | 40 | 19812400, 21565290, 20728471, 22723983, 22479552, 18431360, 20033209 |
| 9 | Hepatitis C virus (HCV) | 278 | 23418453,17943132, 21070682,20969775, 21821155,19144437, 23824794,12624257, WO2011/109380A1, 19524505,18521080, 16141076 |
| 11 | Herpes simplex virus (HSV) | 1 | 21291913 |
| 10 | Human cytomegalovirus (HCMV) | 4 | 20643939 |
| 12 | Human immunodeficiency virus-1 (HIV-1) | 38 | 16236258,22080513, 17906637,19148268, 21224041,17322031, 23042677,17604727, 12007417,24932481, 22709537,17906637, 22112720,19102781 |
| 13 | Human papillomavirus (HPV) | 1 | 20736742 |
| 14 | Infectious bursal disease virus (IBDV) | 7 | 22238234 |
| 15 | Influenza A virus (INFV) | 119 | 22606348, 20554777, WO/2010/101663 A2 |
| 16 | Kaposi sarcoma-associated herpesvirus (KSHV) | 1 | 20081837 |
| 17 | Mink enteritis virus (MEV) | 1 | 24349084 |
| 18 | Mouse cytomegalovirus (MCMV) | 2 | 20047990 |
| 19 | Porcine Reproductive and Respiratory Syndrome Virus (PRRSV) | 3 | 23740977, 24878990, 23409058 |
| 20 | Primate foamy virus (PFV) | 2 | 15845854 |
| 21 | Respiratory syncytial virus (RSV) | 1 | 22272270 |
| 22 | Simian immunodeficiency virus (SIV) | 4 | 23988154 |
| 23 | Varicella-zoster virus (VZV) | 3 | 22676898, 20643939, 18700235, 18668040 |
| 24 | Vesicular stomatitis virus (VSV) | 21 | 22676898, 21431677, 20643939, 20937844 |

**Table S4.** Host genes targeted by multiple viral miRNAs in VIRmiRtar sub-database

| **S. No.** | **Target** | **Viral miRNAs** | **No. of miRNA** | **Description** | **UniProt ID** | **Reference** |
| --- | --- | --- | --- | --- | --- | --- |
| 1 | CCNT2 | kshv-miR-k12-7, ebv-miR-bart9, ebv-miR-bart1-3p, ebv-miR-bart10, kshv-miR-k12-5, kshv-miR-k12-1 | 6 | Cyclin-T2 | F2Z2C9 | 22100165, 22291592, 22473208 |
| 2 | BRWD1 | kshv-miR-k12-11, kshv-miR-k12-6-3p, ebv-miR-bart6-3p, ebv-miR-bart4, ebv-miR-bart3 | 5 | Bromodomain and WD repeat-containing protein 1 | Q9NSI6 | 22100165, 22291592 |
| 3 | BTBD3 | ebv-miR-bart8*, kshv-miR-k12-9, ebv-miR-bart10, kshv-miR-k12-5, ebv-miR-bart15 | 5 | BTB/POZ domain-containing protein 3 | F8WAQ4 | 22100165 |
| 4 | FNDC3A | kshv-miR-k12-5, ebv-miR-bart6-3p, ebv-miR-bart2-5p, ebv-miR-bart14, ebv-miR-bart3 | 5 | Fibronectin type III domain containing 3A, isoform CRA_f | G5E9X3 | 22100165, 22291592 |
| 5 | LCOR | kshv-miR-k12-10b, kshv-miR-k12-4-3p, ebv-miR-bart9*, ebv-miR-bart19-3p, ebv-miR-bart4 | 5 | Ligand-dependent corepressor | Q96JN0 | 22100165, 22291592 |
| 6 | LMBR1 | kshv-miR-k12-9*, ebv-miR-bart20-3p, kshv-miR-k12-6-3p, ebv-miR-bart3*, ebv-miR-bart15 | 5 | Limb region 1 protein homolog | F8WDW0 | 22100165, 22291592 |
| 7 | MEQ | mdv1-miR-m1, mdv1-miR-m2-5p, mdv1-miR-m3, mdv1-miR-m4, mdv1-miR-m5 | 5 | Oncoprotein MEQ | Q9DGW5 | 16912324 |
| 8 | MLL | kshv-miR-k12-9, ebv-miR-bart6-5p, ebv-miR-bart10, kshv-miR-k12-1, ebv-miR-bart19-3p | 5 | Histone-lysine N-methyltransferase 2A | Q03164 | 22100165, 22473208 |
| 9 | NCOR1 | kshv-miR-k12-12, ebv-miR-bart8, ebv-miR-bart20-3p, kshv-miR-k12-1, ebv-miR-bart16 | 5 | Nuclear receptor corepressor 1 | O75376 | 22100165 |
| 10 | PRPF40A | kshv-miR-k12-10a, ebv-miR-bart19-3p, kshv-miR-k12-2, ebv-miR-bart14, ebv-miR-bhrf1-2 | 5 | Pre-mRNA-processing factor 40 homolog A | H7BXZ7 | 22100165, 22291592 |
| 11 | STAG2 | kshv-miR-k12-6-3p, kshv-miR-k12-11, kshv-miR-k12-11, ebv-miR-bart19-3p, ebv-miR-bhrf1-1 | 5 | Cohesin subunit SA-2 | Q8N3U4 | 22100165, 22291592 |
| 12 | TNRC6A | kshv-miR-k12-4-5p, kshv-miR-k12-11*, ebv-miR-bart15, ebv-miR-bart22, ebv-miR-bart18-5p | 5 | Trinucleotide repeat-containing gene 6A protein | Q8NDV7 | 22100165, 25012295 |
| 13 | UBN2 | kshv-miR-k12-8*, kshv-miR-k12-4-3p, ebv-miR-bart8*, ebv-miR-bart18-5p, ebv-miR-bart1-5p | 5 | Ubinuclein-2 | Q6ZU65 | 22100165, 22291592 |
| 14 | ZNF451 | kshv-miR-k12-4-3p, kshv-miR-k12-6-3p, ebv-miR-bart10, ebv-miR-bart5, ebv-miR-bhrf1-2 | 5 | Zinc finger protein 451 | Q9Y4E5 | 22100165, 22291592 |
| 15 | ARHGAP11A | ebv-miR-bart7, kshv-miR-k12-5, ebv-miR-bart22, ebv-miR-bart3 | 4 | Rho GTPase-activating protein 11A | Q6P4F7 | 22473208, 22100165, 22291592 |
| 16 | ARMC8 | ebv-miR-bart10, ebv-miR-bart19-5p, ebv-miR-bart21-3p, ebv-miR-bart5 | 4 | Armadillo repeat-containing protein 8 | Q8IUR7 | 22473208, 22100165 |
| 17 | ATF2 | kshv-miR-k12-10b, ebv-miR-bart17-5p, kshv-miR-k12-2, ebv-miR-bart2-5p | 4 | Cyclic AMP-dependent transcription factor ATF-2 | F2Z2K2 | 22100165 |
| 18 | ATL2 | ebv-miR-bart12, ebv-miR-bart18-5p, ebv-miR-bart22, kshv-miR-k12-1 | 4 | Atlastin-2 | B5MCN0 | 22100165 |
| 19 | C11orf30 | kshv-miR-k12-6-3p, ebv-miR-bart9*, kshv-miR-k12-11, ebv-miR-bart14 | 4 | Protein EMSY | Q7Z589 | 22100165 |
| 20 | C13orf23 | kshv-miR-k12-10b, kshv-miR-k12-6-5p, ebv-miR-bart8, ebv-miR-bart4 | 4 | Proline and serine-rich protein 1 | Q86XN7 | 22100165, 22291592 |

**Table S5.**  AVIRmiRNAs and their multiple viral genes

| **S.No.** | **miRNA name** | **No. of Targets** | **Target name** | **Virus Name** | **Biological Process involved** | **Reference** |
| --- | --- | --- | --- | --- | --- | --- |
| 1 | hsa-miR-181b-5p | 10 | CD163, M1, M2, NA, NEP, NP, NS1, PA, PB1, PB2 | INFV, VZV, PRRSV | Replication | WO/2010/101663/A, 23740977, 22676898, 20643945, 20643939 |
| 2 | hsa-let-7 | 10 | Casp3, DICER1, HMGA2, IFN-beta, IGF2BP1, IGF2BP2, MYC, RAB40C, STAT3 | HBV, VZV, HCV | Replication, Translation, Interferon system | 21565290, 18668040, 23824794, 20969775, 18700235, 20643939 |
| 3 | hsa-miR-142-5p | 9 | M1, M2, NA, NEP, NP, NS1, PA, PB1, PB2, 3' UTR | INFV, DENV | Replication | 22241991, WO/2010/101663/A2 |
| 4 | hsa-miR-149 | 9 | M1, M2, NEP, NP, NS1, PA, PB1, PB2, vpr | HIV, INFV | Replication, Translation | 16236258, WO/2010/101663/A2 |
| 5 | hsa-miR-16 | 9 | M1, M2, Nef, NEP, NP, NS1, PA, PB1, PB2 | INFV, HIV | Replication | 22080513, WO/2010/101663/A2 |
| 6 | hsa-miR-17-5p | 9 | M1, M2, Nef, NEP, NP, NS1, PA, PB1, PB2 | INFV, HIV | Replication | 17322031, WO/2010/101663/A2 |
| 7 | hsa-miR-93 | 9 | IRES, M1, M2, NEP, NP, NS1, PA, PB1, PB2 | INFV, VSV | Replication | 21431677, WO/2010/101663/A2 |
| 8 | hsa-miR-122 | 8 | 5' UTR, CAT-1, CCNG1, HBsAg, HO-1, IFN-beta, NDRG3, SOX6 | VZV, BDV, HCV | Replication, Translation | 20561966, 18431360, 21725618, 21565290, 21821155 |
| 9 | hsa-miR-1259 | 8 | M1, M2, NEP, NP, NS1, PA, PB1, PB2 | INFV | Replication, Interferon system | 22676898, 20643939, 18431360, 20561966, 21565290, 21821155, 16141076 |
| 10 | hsa-miR-1977 | 8 | M1, M2, NEP, NP, NS1, PB1, PB2, PA | INFV | Replication | WO/2010/101663/A2 |
| 11 | hsa-miR-1978 | 8 | M1, M2, NEP, NP, NS1, PB1, PB2, PA | INFV | Replication | WO/2010/101663/A2 |
| 12 | hsa-miR-19a | 8 | M1, M2, NEP, NP, NS1, PB1, PB2, PA | INFV | Replication | WO/2010/101663/A2 |
| 13 | hsa-miR-222 | 8 | M1, M2, NEP, NP, NS1, PB1, PB2, PA | INFV | Replication | WO/2010/101663/A2 |
| 14 | hsa-miR-25-5p | 8 | M1, M2, NEP, NP, NS1, PB1, PB2, PA | INFV | Replication | WO/2010/101663/A2 |
| 15 | hsa-miR-34 | 8 | M1, M2, NEP, NP, NS1, PB1, PB2, PA | INFV | Replication, Interferon system | WO/2010/101663/A221565290, 20969775, 12624257 |
| 16 | hsa-miR-92a | 8 | M1, M2, NEP, NP, NS1, PB1, PB2, PA | INFV | Replication | WO/2010/101663/A2 |
| 17 | hsa-miR-1 | 3 | E2F5, IFN-beta, HDAC4, | HBV | Replication, Interferon system | 21565290, 17943132, 21070682 |
| 18 | hsa-miR-1254 | 4 | CPE, DPP3, MST1, PRSS12 | INFV | Replication | 22606348 |
| 19 | hsa-miR-221 | 4 | Bmf, CDKN1A, DDIT4, NGF | RSV, HBV, HCV | Replication, Translation | 21565290, 21821155, 22272270 |
| 20 | hsa-miR-296-5p | 3 | IFN-beta, VP1, VP3, | EV71, HCV | Replication, Interferon system | 23468506, 17943132, 21070682 |
