# Supplementary material for: VIRmiRNA: a comprehensive resource for experimentally validated viral miRNAs and their targets
Source: Database (Oxford). 2014 Nov 6;2014:bau103. doi: 10.1093/database/bau103 (PMC4224276; doi:10.1093/database/bau103)
Supplement: Supplementary Data [file supp_bau103_Supplementary_2_R2.doc]

**Supplementary Material**

**Supplementary File 2**

Pathway enrichment analysis performed on the target genes of VIRmiRtar sub-database using KEGG Mapper – Search Pathway

[hsa01100 Metabolic pathways - Homo sapiens (human)](http://www.genome.jp/kegg-bin/show_pathway?140722978011892/hsa01100.args) ([188](javascript:display('hsa01100')))

[hsa05200 Pathways in cancer - Homo sapiens (human)](http://www.genome.jp/kegg-bin/show_pathway?140722978011892/hsa05200.args) ([96](javascript:display('hsa05200')))

[hsa05203 Viral carcinogenesis - Homo sapiens (human)](http://www.genome.jp/kegg-bin/show_pathway?140722978011892/hsa05203.args) ([84](javascript:display('hsa05203')))

[hsa05166 HTLV-I infection - Homo sapiens (human)](http://www.genome.jp/kegg-bin/show_pathway?140722978011892/hsa05166.args) ([78](javascript:display('hsa05166')))

[hsa04151 PI3K-Akt signaling pathway - Homo sapiens (human)](http://www.genome.jp/kegg-bin/show_pathway?140722978011892/hsa04151.args) ([69](javascript:display('hsa04151')))

[hsa05206 MicroRNAs in cancer - Homo sapiens (human)](http://www.genome.jp/kegg-bin/show_pathway?140722978011892/hsa05206.args) ([62](javascript:display('hsa05206')))

[hsa05034 Alcoholism - Homo sapiens (human)](http://www.genome.jp/kegg-bin/show_pathway?140722978011892/hsa05034.args) ([62](javascript:display('hsa05034')))

[hsa05169 Epstein-Barr virus infection - Homo sapiens (human)](http://www.genome.jp/kegg-bin/show_pathway?140722978011892/hsa05169.args) ([62](javascript:display('hsa05169')))

[hsa04144 Endocytosis - Homo sapiens (human)](http://www.genome.jp/kegg-bin/show_pathway?140722978011892/hsa04144.args) ([61](javascript:display('hsa04144')))

[hsa04010 MAPK signaling pathway - Homo sapiens (human)](http://www.genome.jp/kegg-bin/show_pathway?140722978011892/hsa04010.args) ([59](javascript:display('hsa04010')))

[hsa05161 Hepatitis B - Homo sapiens (human)](http://www.genome.jp/kegg-bin/show_pathway?140722978011892/hsa05161.args) ([57](javascript:display('hsa05161')))

[hsa04141 Protein processing in endoplasmic reticulum - Homo sapiens (human)](http://www.genome.jp/kegg-bin/show_pathway?140722978011892/hsa04141.args) ([56](javascript:display('hsa04141')))

[hsa05205 Proteoglycans in cancer - Homo sapiens (human)](http://www.genome.jp/kegg-bin/show_pathway?140722978011892/hsa05205.args) ([54](javascript:display('hsa05205')))

[hsa05168 Herpes simplex infection - Homo sapiens (human)](http://www.genome.jp/kegg-bin/show_pathway?140722978011892/hsa05168.args) ([51](javascript:display('hsa05168')))

[hsa05202 Transcriptional misregulation in cancer - Homo sapiens (human)](http://www.genome.jp/kegg-bin/show_pathway?140722978011892/hsa05202.args) ([51](javascript:display('hsa05202')))

[hsa04510 Focal adhesion - Homo sapiens (human)](http://www.genome.jp/kegg-bin/show_pathway?140722978011892/hsa04510.args) ([49](javascript:display('hsa04510')))

[hsa05162 Measles - Homo sapiens (human)](http://www.genome.jp/kegg-bin/show_pathway?140722978011892/hsa05162.args) ([48](javascript:display('hsa05162')))

[hsa05152 Tuberculosis - Homo sapiens (human)](http://www.genome.jp/kegg-bin/show_pathway?140722978011892/hsa05152.args) ([47](javascript:display('hsa05152')))

[hsa04390 Hippo signaling pathway - Homo sapiens (human)](http://www.genome.jp/kegg-bin/show_pathway?140722978011892/hsa04390.args) ([47](javascript:display('hsa04390')))

[hsa04068 FoxO signaling pathway - Homo sapiens (human)](http://www.genome.jp/kegg-bin/show_pathway?140722978011892/hsa04068.args) ([47](javascript:display('hsa04068')))

[hsa04110 Cell cycle - Homo sapiens (human)](http://www.genome.jp/kegg-bin/show_pathway?140722978011892/hsa04110.args) ([47](javascript:display('hsa04110')))

[hsa04024 cAMP signaling pathway - Homo sapiens (human)](http://www.genome.jp/kegg-bin/show_pathway?140722978011892/hsa04024.args) ([46](javascript:display('hsa04024')))

[hsa05322 Systemic lupus erythematosus - Homo sapiens (human)](http://www.genome.jp/kegg-bin/show_pathway?140722978011892/hsa05322.args) ([45](javascript:display('hsa05322')))

[hsa05164 Influenza A - Homo sapiens (human)](http://www.genome.jp/kegg-bin/show_pathway?140722978011892/hsa05164.args) ([45](javascript:display('hsa05164')))

[hsa04014 Ras signaling pathway - Homo sapiens (human)](http://www.genome.jp/kegg-bin/show_pathway?140722978011892/hsa04014.args) ([45](javascript:display('hsa04014')))

[hsa03013 RNA transport - Homo sapiens (human)](http://www.genome.jp/kegg-bin/show_pathway?140722978011892/hsa03013.args) ([45](javascript:display('hsa03013')))

[hsa04015 Rap1 signaling pathway - Homo sapiens (human)](http://www.genome.jp/kegg-bin/show_pathway?140722978011892/hsa04015.args) ([43](javascript:display('hsa04015')))

[hsa04910 Insulin signaling pathway - Homo sapiens (human)](http://www.genome.jp/kegg-bin/show_pathway?140722978011892/hsa04910.args) ([43](javascript:display('hsa04910')))

[hsa04722 Neurotrophin signaling pathway - Homo sapiens (human)](http://www.genome.jp/kegg-bin/show_pathway?140722978011892/hsa04722.args) ([43](javascript:display('hsa04722')))

[hsa04022 cGMP-PKG signaling pathway - Homo sapiens (human)](http://www.genome.jp/kegg-bin/show_pathway?140722978011892/hsa04022.args) ([43](javascript:display('hsa04022')))

[hsa04310 Wnt signaling pathway - Homo sapiens (human)](http://www.genome.jp/kegg-bin/show_pathway?140722978011892/hsa04310.args) ([42](javascript:display('hsa04310')))

[hsa04152 AMPK signaling pathway - Homo sapiens (human)](http://www.genome.jp/kegg-bin/show_pathway?140722978011892/hsa04152.args) ([42](javascript:display('hsa04152')))

[hsa05016 Huntington's disease - Homo sapiens (human)](http://www.genome.jp/kegg-bin/show_pathway?140722978011892/hsa05016.args) ([42](javascript:display('hsa05016')))

[hsa04810 Regulation of actin cytoskeleton - Homo sapiens (human)](http://www.genome.jp/kegg-bin/show_pathway?140722978011892/hsa04810.args) ([41](javascript:display('hsa04810')))

[hsa04919 Thyroid hormone signaling pathway - Homo sapiens (human)](http://www.genome.jp/kegg-bin/show_pathway?140722978011892/hsa04919.args) ([41](javascript:display('hsa04919')))

[hsa04921 Oxytocin signaling pathway - Homo sapiens (human)](http://www.genome.jp/kegg-bin/show_pathway?140722978011892/hsa04921.args) ([40](javascript:display('hsa04921')))

[hsa04114 Oocyte meiosis - Homo sapiens (human)](http://www.genome.jp/kegg-bin/show_pathway?140722978011892/hsa04114.args) ([40](javascript:display('hsa04114')))

[hsa04062 Chemokine signaling pathway - Homo sapiens (human)](http://www.genome.jp/kegg-bin/show_pathway?140722978011892/hsa04062.args) ([40](javascript:display('hsa04062')))

[hsa00230 Purine metabolism - Homo sapiens (human)](http://www.genome.jp/kegg-bin/show_pathway?140722978011892/hsa00230.args) ([38](javascript:display('hsa00230')))

[hsa04261 Adrenergic signaling in cardiomyocytes - Homo sapiens (human)](http://www.genome.jp/kegg-bin/show_pathway?140722978011892/hsa04261.args) ([37](javascript:display('hsa04261')))

[hsa04120 Ubiquitin mediated proteolysis - Homo sapiens (human)](http://www.genome.jp/kegg-bin/show_pathway?140722978011892/hsa04120.args) ([37](javascript:display('hsa04120')))

[hsa03040 Spliceosome - Homo sapiens (human)](http://www.genome.jp/kegg-bin/show_pathway?140722978011892/hsa03040.args) ([37](javascript:display('hsa03040')))

[hsa05160 Hepatitis C - Homo sapiens (human)](http://www.genome.jp/kegg-bin/show_pathway?140722978011892/hsa05160.args) ([37](javascript:display('hsa05160')))

[hsa04630 Jak-STAT signaling pathway - Homo sapiens (human)](http://www.genome.jp/kegg-bin/show_pathway?140722978011892/hsa04630.args) ([36](javascript:display('hsa04630')))

[hsa05215 Prostate cancer - Homo sapiens (human)](http://www.genome.jp/kegg-bin/show_pathway?140722978011892/hsa05215.args) ([36](javascript:display('hsa05215')))

[hsa05220 Chronic myeloid leukemia - Homo sapiens (human)](http://www.genome.jp/kegg-bin/show_pathway?140722978011892/hsa05220.args) ([36](javascript:display('hsa05220')))

[hsa04932 Non-alcoholic fatty liver disease (NAFLD) - Homo sapiens (human)](http://www.genome.jp/kegg-bin/show_pathway?140722978011892/hsa04932.args) ([35](javascript:display('hsa04932')))

[hsa05010 Alzheimer's disease - Homo sapiens (human)](http://www.genome.jp/kegg-bin/show_pathway?140722978011892/hsa05010.args) ([34](javascript:display('hsa05010')))

[hsa05145 Toxoplasmosis - Homo sapiens (human)](http://www.genome.jp/kegg-bin/show_pathway?140722978011892/hsa05145.args) ([34](javascript:display('hsa05145')))

[hsa03015 mRNA surveillance pathway - Homo sapiens (human)](http://www.genome.jp/kegg-bin/show_pathway?140722978011892/hsa03015.args) ([34](javascript:display('hsa03015')))

[hsa04660 T cell receptor signaling pathway - Homo sapiens (human)](http://www.genome.jp/kegg-bin/show_pathway?140722978011892/hsa04660.args) ([34](javascript:display('hsa04660')))

[hsa04380 Osteoclast differentiation - Homo sapiens (human)](http://www.genome.jp/kegg-bin/show_pathway?140722978011892/hsa04380.args) ([33](javascript:display('hsa04380')))

[hsa04142 Lysosome - Homo sapiens (human)](http://www.genome.jp/kegg-bin/show_pathway?140722978011892/hsa04142.args) ([33](javascript:display('hsa04142')))

[hsa04668 TNF signaling pathway - Homo sapiens (human)](http://www.genome.jp/kegg-bin/show_pathway?140722978011892/hsa04668.args) ([33](javascript:display('hsa04668')))

[hsa04210 Apoptosis - Homo sapiens (human)](http://www.genome.jp/kegg-bin/show_pathway?140722978011892/hsa04210.args) ([32](javascript:display('hsa04210')))

[hsa04916 Melanogenesis - Homo sapiens (human)](http://www.genome.jp/kegg-bin/show_pathway?140722978011892/hsa04916.args) ([32](javascript:display('hsa04916')))

[hsa04115 p53 signaling pathway - Homo sapiens (human)](http://www.genome.jp/kegg-bin/show_pathway?140722978011892/hsa04115.args) ([32](javascript:display('hsa04115')))

[hsa05222 Small cell lung cancer - Homo sapiens (human)](http://www.genome.jp/kegg-bin/show_pathway?140722978011892/hsa05222.args) ([31](javascript:display('hsa05222')))

[hsa04915 Estrogen signaling pathway - Homo sapiens (human)](http://www.genome.jp/kegg-bin/show_pathway?140722978011892/hsa04915.args) ([31](javascript:display('hsa04915')))

[hsa04728 Dopaminergic synapse - Homo sapiens (human)](http://www.genome.jp/kegg-bin/show_pathway?140722978011892/hsa04728.args) ([31](javascript:display('hsa04728')))

[hsa04145 Phagosome - Homo sapiens (human)](http://www.genome.jp/kegg-bin/show_pathway?140722978011892/hsa04145.args) ([30](javascript:display('hsa04145')))

[hsa05210 Colorectal cancer - Homo sapiens (human)](http://www.genome.jp/kegg-bin/show_pathway?140722978011892/hsa05210.args) ([30](javascript:display('hsa05210')))

[hsa05212 Pancreatic cancer - Homo sapiens (human)](http://www.genome.jp/kegg-bin/show_pathway?140722978011892/hsa05212.args) ([30](javascript:display('hsa05212')))

[hsa04012 ErbB signaling pathway - Homo sapiens (human)](http://www.genome.jp/kegg-bin/show_pathway?140722978011892/hsa04012.args) ([29](javascript:display('hsa04012')))

[hsa04070 Phosphatidylinositol signaling system - Homo sapiens (human)](http://www.genome.jp/kegg-bin/show_pathway?140722978011892/hsa04070.args) ([29](javascript:display('hsa04070')))

[hsa04520 Adherens junction - Homo sapiens (human)](http://www.genome.jp/kegg-bin/show_pathway?140722978011892/hsa04520.args) ([29](javascript:display('hsa04520')))

[hsa04611 Platelet activation - Homo sapiens (human)](http://www.genome.jp/kegg-bin/show_pathway?140722978011892/hsa04611.args) ([29](javascript:display('hsa04611')))

[hsa04917 Prolactin signaling pathway - Homo sapiens (human)](http://www.genome.jp/kegg-bin/show_pathway?140722978011892/hsa04917.args) ([28](javascript:display('hsa04917')))

[hsa03018 RNA degradation - Homo sapiens (human)](http://www.genome.jp/kegg-bin/show_pathway?140722978011892/hsa03018.args) ([28](javascript:display('hsa03018')))

[hsa04066 HIF-1 signaling pathway - Homo sapiens (human)](http://www.genome.jp/kegg-bin/show_pathway?140722978011892/hsa04066.args) ([28](javascript:display('hsa04066')))

[hsa05214 Glioma - Homo sapiens (human)](http://www.genome.jp/kegg-bin/show_pathway?140722978011892/hsa05214.args) ([27](javascript:display('hsa05214')))

[hsa05142 Chagas disease (American trypanosomiasis) - Homo sapiens (human)](http://www.genome.jp/kegg-bin/show_pathway?140722978011892/hsa05142.args) ([27](javascript:display('hsa05142')))

[hsa00240 Pyrimidine metabolism - Homo sapiens (human)](http://www.genome.jp/kegg-bin/show_pathway?140722978011892/hsa00240.args) ([26](javascript:display('hsa00240')))

[hsa04725 Cholinergic synapse - Homo sapiens (human)](http://www.genome.jp/kegg-bin/show_pathway?140722978011892/hsa04725.args) ([26](javascript:display('hsa04725')))

[hsa04650 Natural killer cell mediated cytotoxicity - Homo sapiens (human)](http://www.genome.jp/kegg-bin/show_pathway?140722978011892/hsa04650.args) ([26](javascript:display('hsa04650')))

[hsa04020 Calcium signaling pathway - Homo sapiens (human)](http://www.genome.jp/kegg-bin/show_pathway?140722978011892/hsa04020.args) ([26](javascript:display('hsa04020')))

[hsa04914 Progesterone-mediated oocyte maturation - Homo sapiens (human)](http://www.genome.jp/kegg-bin/show_pathway?140722978011892/hsa04914.args) ([26](javascript:display('hsa04914')))

[hsa04662 B cell receptor signaling pathway - Homo sapiens (human)](http://www.genome.jp/kegg-bin/show_pathway?140722978011892/hsa04662.args) ([26](javascript:display('hsa04662')))

[hsa04620 Toll-like receptor signaling pathway - Homo sapiens (human)](http://www.genome.jp/kegg-bin/show_pathway?140722978011892/hsa04620.args) ([25](javascript:display('hsa04620')))

[hsa05211 Renal cell carcinoma - Homo sapiens (human)](http://www.genome.jp/kegg-bin/show_pathway?140722978011892/hsa05211.args) ([25](javascript:display('hsa05211')))

[hsa04360 Axon guidance - Homo sapiens (human)](http://www.genome.jp/kegg-bin/show_pathway?140722978011892/hsa04360.args) ([25](javascript:display('hsa04360')))

[hsa05213 Endometrial cancer - Homo sapiens (human)](http://www.genome.jp/kegg-bin/show_pathway?140722978011892/hsa05213.args) ([24](javascript:display('hsa05213')))

[hsa04530 Tight junction - Homo sapiens (human)](http://www.genome.jp/kegg-bin/show_pathway?140722978011892/hsa04530.args) ([24](javascript:display('hsa04530')))

[hsa05221 Acute myeloid leukemia - Homo sapiens (human)](http://www.genome.jp/kegg-bin/show_pathway?140722978011892/hsa05221.args) ([23](javascript:display('hsa05221')))

[hsa04912 GnRH signaling pathway - Homo sapiens (human)](http://www.genome.jp/kegg-bin/show_pathway?140722978011892/hsa04912.args) ([23](javascript:display('hsa04912')))

[hsa05223 Non-small cell lung cancer - Homo sapiens (human)](http://www.genome.jp/kegg-bin/show_pathway?140722978011892/hsa05223.args) ([23](javascript:display('hsa05223')))

[hsa04666 Fc gamma R-mediated phagocytosis - Homo sapiens (human)](http://www.genome.jp/kegg-bin/show_pathway?140722978011892/hsa04666.args) ([22](javascript:display('hsa04666')))

[hsa05218 Melanoma - Homo sapiens (human)](http://www.genome.jp/kegg-bin/show_pathway?140722978011892/hsa05218.args) ([22](javascript:display('hsa05218')))

[hsa04270 Vascular smooth muscle contraction - Homo sapiens (human)](http://www.genome.jp/kegg-bin/show_pathway?140722978011892/hsa04270.args) ([22](javascript:display('hsa04270')))

[hsa04540 Gap junction - Homo sapiens (human)](http://www.genome.jp/kegg-bin/show_pathway?140722978011892/hsa04540.args) ([22](javascript:display('hsa04540')))

[hsa00564 Glycerophospholipid metabolism - Homo sapiens (human)](http://www.genome.jp/kegg-bin/show_pathway?140722978011892/hsa00564.args) ([22](javascript:display('hsa00564')))

[hsa04670 Leukocyte transendothelial migration - Homo sapiens (human)](http://www.genome.jp/kegg-bin/show_pathway?140722978011892/hsa04670.args) ([21](javascript:display('hsa04670')))

[hsa00190 Oxidative phosphorylation - Homo sapiens (human)](http://www.genome.jp/kegg-bin/show_pathway?140722978011892/hsa00190.args) ([21](javascript:display('hsa00190')))

[hsa04150 mTOR signaling pathway - Homo sapiens (human)](http://www.genome.jp/kegg-bin/show_pathway?140722978011892/hsa04150.args) ([20](javascript:display('hsa04150')))

[hsa05031 Amphetamine addiction - Homo sapiens (human)](http://www.genome.jp/kegg-bin/show_pathway?140722978011892/hsa05031.args) ([20](javascript:display('hsa05031')))

[hsa04720 Long-term potentiation - Homo sapiens (human)](http://www.genome.jp/kegg-bin/show_pathway?140722978011892/hsa04720.args) ([20](javascript:display('hsa04720')))

[hsa04064 NF-kappa B signaling pathway - Homo sapiens (human)](http://www.genome.jp/kegg-bin/show_pathway?140722978011892/hsa04064.args) ([20](javascript:display('hsa04064')))

[hsa04713 Circadian entrainment - Homo sapiens (human)](http://www.genome.jp/kegg-bin/show_pathway?140722978011892/hsa04713.args) ([20](javascript:display('hsa04713')))

[hsa04920 Adipocytokine signaling pathway - Homo sapiens (human)](http://www.genome.jp/kegg-bin/show_pathway?140722978011892/hsa04920.args) ([20](javascript:display('hsa04920')))

[hsa05100 Bacterial invasion of epithelial cells - Homo sapiens (human)](http://www.genome.jp/kegg-bin/show_pathway?140722978011892/hsa05100.args) ([20](javascript:display('hsa05100')))

[hsa04750 Inflammatory mediator regulation of TRP channels - Homo sapiens (human)](http://www.genome.jp/kegg-bin/show_pathway?140722978011892/hsa04750.args) ([20](javascript:display('hsa04750')))

[hsa04350 TGF-beta signaling pathway - Homo sapiens (human)](http://www.genome.jp/kegg-bin/show_pathway?140722978011892/hsa04350.args) ([19](javascript:display('hsa04350')))

[hsa04060 Cytokine-cytokine receptor interaction - Homo sapiens (human)](http://www.genome.jp/kegg-bin/show_pathway?140722978011892/hsa04060.args) ([19](javascript:display('hsa04060')))

[hsa05146 Amoebiasis - Homo sapiens (human)](http://www.genome.jp/kegg-bin/show_pathway?140722978011892/hsa05146.args) ([19](javascript:display('hsa05146')))

[hsa00562 Inositol phosphate metabolism - Homo sapiens (human)](http://www.genome.jp/kegg-bin/show_pathway?140722978011892/hsa00562.args) ([19](javascript:display('hsa00562')))

[hsa05012 Parkinson's disease - Homo sapiens (human)](http://www.genome.jp/kegg-bin/show_pathway?140722978011892/hsa05012.args) ([19](javascript:display('hsa05012')))

[hsa04724 Glutamatergic synapse - Homo sapiens (human)](http://www.genome.jp/kegg-bin/show_pathway?140722978011892/hsa04724.args) ([19](javascript:display('hsa04724')))

[hsa04146 Peroxisome - Homo sapiens (human)](http://www.genome.jp/kegg-bin/show_pathway?140722978011892/hsa04146.args) ([18](javascript:display('hsa04146')))

[hsa05110 Vibrio cholerae infection - Homo sapiens (human)](http://www.genome.jp/kegg-bin/show_pathway?140722978011892/hsa05110.args) ([18](javascript:display('hsa05110')))

[hsa04370 VEGF signaling pathway - Homo sapiens (human)](http://www.genome.jp/kegg-bin/show_pathway?140722978011892/hsa04370.args) ([18](javascript:display('hsa04370')))

[hsa04970 Salivary secretion - Homo sapiens (human)](http://www.genome.jp/kegg-bin/show_pathway?140722978011892/hsa04970.args) ([18](javascript:display('hsa04970')))

[hsa04622 RIG-I-like receptor signaling pathway - Homo sapiens (human)](http://www.genome.jp/kegg-bin/show_pathway?140722978011892/hsa04622.args) ([18](javascript:display('hsa04622')))

[hsa05130 Pathogenic Escherichia coli infection - Homo sapiens (human)](http://www.genome.jp/kegg-bin/show_pathway?140722978011892/hsa05130.args) ([17](javascript:display('hsa05130')))

[hsa04971 Gastric acid secretion - Homo sapiens (human)](http://www.genome.jp/kegg-bin/show_pathway?140722978011892/hsa04971.args) ([17](javascript:display('hsa04971')))

[hsa04330 Notch signaling pathway - Homo sapiens (human)](http://www.genome.jp/kegg-bin/show_pathway?140722978011892/hsa04330.args) ([17](javascript:display('hsa04330')))

[hsa04664 Fc epsilon RI signaling pathway - Homo sapiens (human)](http://www.genome.jp/kegg-bin/show_pathway?140722978011892/hsa04664.args) ([16](javascript:display('hsa04664')))

[hsa01200 Carbon metabolism - Homo sapiens (human)](http://www.genome.jp/kegg-bin/show_pathway?140722978011892/hsa01200.args) ([16](javascript:display('hsa01200')))

[hsa05133 Pertussis - Homo sapiens (human)](http://www.genome.jp/kegg-bin/show_pathway?140722978011892/hsa05133.args) ([16](javascript:display('hsa05133')))

[hsa00310 Lysine degradation - Homo sapiens (human)](http://www.genome.jp/kegg-bin/show_pathway?140722978011892/hsa00310.args) ([16](javascript:display('hsa00310')))

[hsa03008 Ribosome biogenesis in eukaryotes - Homo sapiens (human)](http://www.genome.jp/kegg-bin/show_pathway?140722978011892/hsa03008.args) ([16](javascript:display('hsa03008')))

[hsa04911 Insulin secretion - Homo sapiens (human)](http://www.genome.jp/kegg-bin/show_pathway?140722978011892/hsa04911.args) ([16](javascript:display('hsa04911')))

[hsa05120 Epithelial cell signaling in Helicobacter pylori infection - Homo sapiens (human)](http://www.genome.jp/kegg-bin/show_pathway?140722978011892/hsa05120.args) ([15](javascript:display('hsa05120')))

[hsa03010 Ribosome - Homo sapiens (human)](http://www.genome.jp/kegg-bin/show_pathway?140722978011892/hsa03010.args) ([15](javascript:display('hsa03010')))

[hsa04621 NOD-like receptor signaling pathway - Homo sapiens (human)](http://www.genome.jp/kegg-bin/show_pathway?140722978011892/hsa04621.args) ([15](javascript:display('hsa04621')))

[hsa05140 Leishmaniasis - Homo sapiens (human)](http://www.genome.jp/kegg-bin/show_pathway?140722978011892/hsa05140.args) ([15](javascript:display('hsa05140')))

[hsa04962 Vasopressin-regulated water reabsorption - Homo sapiens (human)](http://www.genome.jp/kegg-bin/show_pathway?140722978011892/hsa04962.args) ([15](javascript:display('hsa04962')))

[hsa04918 Thyroid hormone synthesis - Homo sapiens (human)](http://www.genome.jp/kegg-bin/show_pathway?140722978011892/hsa04918.args) ([15](javascript:display('hsa04918')))

[hsa05131 Shigellosis - Homo sapiens (human)](http://www.genome.jp/kegg-bin/show_pathway?140722978011892/hsa05131.args) ([15](javascript:display('hsa05131')))

[hsa05219 Bladder cancer - Homo sapiens (human)](http://www.genome.jp/kegg-bin/show_pathway?140722978011892/hsa05219.args) ([15](javascript:display('hsa05219')))

[hsa05132 Salmonella infection - Homo sapiens (human)](http://www.genome.jp/kegg-bin/show_pathway?140722978011892/hsa05132.args) ([14](javascript:display('hsa05132')))

[hsa05014 Amyotrophic lateral sclerosis (ALS) - Homo sapiens (human)](http://www.genome.jp/kegg-bin/show_pathway?140722978011892/hsa05014.args) ([14](javascript:display('hsa05014')))

[hsa04612 Antigen processing and presentation - Homo sapiens (human)](http://www.genome.jp/kegg-bin/show_pathway?140722978011892/hsa04612.args) ([14](javascript:display('hsa04612')))

[hsa04727 GABAergic synapse - Homo sapiens (human)](http://www.genome.jp/kegg-bin/show_pathway?140722978011892/hsa04727.args) ([14](javascript:display('hsa04727')))

[hsa05134 Legionellosis - Homo sapiens (human)](http://www.genome.jp/kegg-bin/show_pathway?140722978011892/hsa05134.args) ([14](javascript:display('hsa05134')))

[hsa04972 Pancreatic secretion - Homo sapiens (human)](http://www.genome.jp/kegg-bin/show_pathway?140722978011892/hsa04972.args) ([14](javascript:display('hsa04972')))

[hsa04514 Cell adhesion molecules (CAMs) - Homo sapiens (human)](http://www.genome.jp/kegg-bin/show_pathway?140722978011892/hsa04514.args) ([14](javascript:display('hsa04514')))

[hsa00280 Valine, leucine and isoleucine degradation - Homo sapiens (human)](http://www.genome.jp/kegg-bin/show_pathway?140722978011892/hsa00280.args) ([13](javascript:display('hsa00280')))

[hsa05217 Basal cell carcinoma - Homo sapiens (human)](http://www.genome.jp/kegg-bin/show_pathway?140722978011892/hsa05217.args) ([13](javascript:display('hsa05217')))

[hsa01212 Fatty acid metabolism - Homo sapiens (human)](http://www.genome.jp/kegg-bin/show_pathway?140722978011892/hsa01212.args) ([13](javascript:display('hsa01212')))

[hsa04723 Retrograde endocannabinoid signaling - Homo sapiens (human)](http://www.genome.jp/kegg-bin/show_pathway?140722978011892/hsa04723.args) ([12](javascript:display('hsa04723')))

[hsa00270 Cysteine and methionine metabolism - Homo sapiens (human)](http://www.genome.jp/kegg-bin/show_pathway?140722978011892/hsa00270.args) ([12](javascript:display('hsa00270')))

[hsa03320 PPAR signaling pathway - Homo sapiens (human)](http://www.genome.jp/kegg-bin/show_pathway?140722978011892/hsa03320.args) ([12](javascript:display('hsa03320')))

[hsa01230 Biosynthesis of amino acids - Homo sapiens (human)](http://www.genome.jp/kegg-bin/show_pathway?140722978011892/hsa01230.args) ([12](javascript:display('hsa01230')))

[hsa05321 Inflammatory bowel disease (IBD) - Homo sapiens (human)](http://www.genome.jp/kegg-bin/show_pathway?140722978011892/hsa05321.args) ([12](javascript:display('hsa05321')))

[hsa04623 Cytosolic DNA-sensing pathway - Homo sapiens (human)](http://www.genome.jp/kegg-bin/show_pathway?140722978011892/hsa04623.args) ([12](javascript:display('hsa04623')))

[hsa00510 N-Glycan biosynthesis - Homo sapiens (human)](http://www.genome.jp/kegg-bin/show_pathway?140722978011892/hsa00510.args) ([12](javascript:display('hsa00510')))

[hsa05414 Dilated cardiomyopathy - Homo sapiens (human)](http://www.genome.jp/kegg-bin/show_pathway?140722978011892/hsa05414.args) ([12](javascript:display('hsa05414')))

[hsa00561 Glycerolipid metabolism - Homo sapiens (human)](http://www.genome.jp/kegg-bin/show_pathway?140722978011892/hsa00561.args) ([12](javascript:display('hsa00561')))

[hsa04976 Bile secretion - Homo sapiens (human)](http://www.genome.jp/kegg-bin/show_pathway?140722978011892/hsa04976.args) ([11](javascript:display('hsa04976')))

[hsa05323 Rheumatoid arthritis - Homo sapiens (human)](http://www.genome.jp/kegg-bin/show_pathway?140722978011892/hsa05323.args) ([11](javascript:display('hsa05323')))

[hsa04726 Serotonergic synapse - Homo sapiens (human)](http://www.genome.jp/kegg-bin/show_pathway?140722978011892/hsa04726.args) ([11](javascript:display('hsa04726')))

[hsa05032 Morphine addiction - Homo sapiens (human)](http://www.genome.jp/kegg-bin/show_pathway?140722978011892/hsa05032.args) ([11](javascript:display('hsa05032')))

[hsa05416 Viral myocarditis - Homo sapiens (human)](http://www.genome.jp/kegg-bin/show_pathway?140722978011892/hsa05416.args) ([11](javascript:display('hsa05416')))

[hsa04730 Long-term depression - Homo sapiens (human)](http://www.genome.jp/kegg-bin/show_pathway?140722978011892/hsa04730.args) ([11](javascript:display('hsa04730')))

[hsa03022 Basal transcription factors - Homo sapiens (human)](http://www.genome.jp/kegg-bin/show_pathway?140722978011892/hsa03022.args) ([10](javascript:display('hsa03022')))

[hsa00071 Fatty acid degradation - Homo sapiens (human)](http://www.genome.jp/kegg-bin/show_pathway?140722978011892/hsa00071.args) ([10](javascript:display('hsa00071')))

[hsa04710 Circadian rhythm - Homo sapiens (human)](http://www.genome.jp/kegg-bin/show_pathway?140722978011892/hsa04710.args) ([10](javascript:display('hsa04710')))

[hsa05216 Thyroid cancer - Homo sapiens (human)](http://www.genome.jp/kegg-bin/show_pathway?140722978011892/hsa05216.args) ([10](javascript:display('hsa05216')))

[hsa03020 RNA polymerase - Homo sapiens (human)](http://www.genome.jp/kegg-bin/show_pathway?140722978011892/hsa03020.args) ([10](javascript:display('hsa03020')))

[hsa04960 Aldosterone-regulated sodium reabsorption - Homo sapiens (human)](http://www.genome.jp/kegg-bin/show_pathway?140722978011892/hsa04960.args) ([10](javascript:display('hsa04960')))

[hsa04930 Type II diabetes mellitus - Homo sapiens (human)](http://www.genome.jp/kegg-bin/show_pathway?140722978011892/hsa04930.args) ([10](javascript:display('hsa04930')))

[hsa04913 Ovarian steroidogenesis - Homo sapiens (human)](http://www.genome.jp/kegg-bin/show_pathway?140722978011892/hsa04913.args) ([9](javascript:display('hsa04913')))

[hsa04973 Carbohydrate digestion and absorption - Homo sapiens (human)](http://www.genome.jp/kegg-bin/show_pathway?140722978011892/hsa04973.args) ([9](javascript:display('hsa04973')))

[hsa05412 Arrhythmogenic right ventricular cardiomyopathy (ARVC) - Homo sapiens (human)](http://www.genome.jp/kegg-bin/show_pathway?140722978011892/hsa05412.args) ([9](javascript:display('hsa05412')))

[hsa04721 Synaptic vesicle cycle - Homo sapiens (human)](http://www.genome.jp/kegg-bin/show_pathway?140722978011892/hsa04721.args) ([9](javascript:display('hsa04721')))

[hsa00970 Aminoacyl-tRNA biosynthesis - Homo sapiens (human)](http://www.genome.jp/kegg-bin/show_pathway?140722978011892/hsa00970.args) ([9](javascript:display('hsa00970')))

[hsa00330 Arginine and proline metabolism - Homo sapiens (human)](http://www.genome.jp/kegg-bin/show_pathway?140722978011892/hsa00330.args) ([9](javascript:display('hsa00330')))

[hsa04130 SNARE interactions in vesicular transport - Homo sapiens (human)](http://www.genome.jp/kegg-bin/show_pathway?140722978011892/hsa04130.args) ([9](javascript:display('hsa04130')))

[hsa04340 Hedgehog signaling pathway - Homo sapiens (human)](http://www.genome.jp/kegg-bin/show_pathway?140722978011892/hsa04340.args) ([8](javascript:display('hsa04340')))

[hsa00010 Glycolysis / Gluconeogenesis - Homo sapiens (human)](http://www.genome.jp/kegg-bin/show_pathway?140722978011892/hsa00010.args) ([8](javascript:display('hsa00010')))

[hsa04080 Neuroactive ligand-receptor interaction - Homo sapiens (human)](http://www.genome.jp/kegg-bin/show_pathway?140722978011892/hsa04080.args) ([8](javascript:display('hsa04080')))

[hsa00520 Amino sugar and nucleotide sugar metabolism - Homo sapiens (human)](http://www.genome.jp/kegg-bin/show_pathway?140722978011892/hsa00520.args) ([8](javascript:display('hsa00520')))

[hsa00600 Sphingolipid metabolism - Homo sapiens (human)](http://www.genome.jp/kegg-bin/show_pathway?140722978011892/hsa00600.args) ([8](javascript:display('hsa00600')))

[hsa04974 Protein digestion and absorption - Homo sapiens (human)](http://www.genome.jp/kegg-bin/show_pathway?140722978011892/hsa04974.args) ([8](javascript:display('hsa04974')))

[hsa00480 Glutathione metabolism - Homo sapiens (human)](http://www.genome.jp/kegg-bin/show_pathway?140722978011892/hsa00480.args) ([8](javascript:display('hsa00480')))

[hsa05030 Cocaine addiction - Homo sapiens (human)](http://www.genome.jp/kegg-bin/show_pathway?140722978011892/hsa05030.args) ([8](javascript:display('hsa05030')))

[hsa05020 Prion diseases - Homo sapiens (human)](http://www.genome.jp/kegg-bin/show_pathway?140722978011892/hsa05020.args) ([8](javascript:display('hsa05020')))

[hsa04260 Cardiac muscle contraction - Homo sapiens (human)](http://www.genome.jp/kegg-bin/show_pathway?140722978011892/hsa04260.args) ([8](javascript:display('hsa04260')))

[hsa04961 Endocrine and other factor-regulated calcium reabsorption - Homo sapiens (human)](http://www.genome.jp/kegg-bin/show_pathway?140722978011892/hsa04961.args) ([8](javascript:display('hsa04961')))

[hsa03060 Protein export - Homo sapiens (human)](http://www.genome.jp/kegg-bin/show_pathway?140722978011892/hsa03060.args) ([7](javascript:display('hsa03060')))

[hsa04966 Collecting duct acid secretion - Homo sapiens (human)](http://www.genome.jp/kegg-bin/show_pathway?140722978011892/hsa04966.args) ([7](javascript:display('hsa04966')))

[hsa03440 Homologous recombination - Homo sapiens (human)](http://www.genome.jp/kegg-bin/show_pathway?140722978011892/hsa03440.args) ([7](javascript:display('hsa03440')))

[hsa00620 Pyruvate metabolism - Homo sapiens (human)](http://www.genome.jp/kegg-bin/show_pathway?140722978011892/hsa00620.args) ([7](javascript:display('hsa00620')))

[hsa01040 Biosynthesis of unsaturated fatty acids - Homo sapiens (human)](http://www.genome.jp/kegg-bin/show_pathway?140722978011892/hsa01040.args) ([7](javascript:display('hsa01040')))

[hsa00250 Alanine, aspartate and glutamate metabolism - Homo sapiens (human)](http://www.genome.jp/kegg-bin/show_pathway?140722978011892/hsa00250.args) ([7](javascript:display('hsa00250')))

[hsa03460 Fanconi anemia pathway - Homo sapiens (human)](http://www.genome.jp/kegg-bin/show_pathway?140722978011892/hsa03460.args) ([7](javascript:display('hsa03460')))

[hsa05410 Hypertrophic cardiomyopathy (HCM) - Homo sapiens (human)](http://www.genome.jp/kegg-bin/show_pathway?140722978011892/hsa05410.args) ([7](javascript:display('hsa05410')))

[hsa03050 Proteasome - Homo sapiens (human)](http://www.genome.jp/kegg-bin/show_pathway?140722978011892/hsa03050.args) ([7](javascript:display('hsa03050')))

[hsa04140 Regulation of autophagy - Homo sapiens (human)](http://www.genome.jp/kegg-bin/show_pathway?140722978011892/hsa04140.args) ([6](javascript:display('hsa04140')))

[hsa00900 Terpenoid backbone biosynthesis - Homo sapiens (human)](http://www.genome.jp/kegg-bin/show_pathway?140722978011892/hsa00900.args) ([6](javascript:display('hsa00900')))

[hsa04512 ECM-receptor interaction - Homo sapiens (human)](http://www.genome.jp/kegg-bin/show_pathway?140722978011892/hsa04512.args) ([6](javascript:display('hsa04512')))

[hsa03420 Nucleotide excision repair - Homo sapiens (human)](http://www.genome.jp/kegg-bin/show_pathway?140722978011892/hsa03420.args) ([6](javascript:display('hsa03420')))

[hsa04320 Dorso-ventral axis formation - Homo sapiens (human)](http://www.genome.jp/kegg-bin/show_pathway?140722978011892/hsa04320.args) ([6](javascript:display('hsa04320')))

[hsa04740 Olfactory transduction - Homo sapiens (human)](http://www.genome.jp/kegg-bin/show_pathway?140722978011892/hsa04740.args) ([6](javascript:display('hsa04740')))

[hsa00020 Citrate cycle (TCA cycle) - Homo sapiens (human)](http://www.genome.jp/kegg-bin/show_pathway?140722978011892/hsa00020.args) ([6](javascript:display('hsa00020')))

[hsa04978 Mineral absorption - Homo sapiens (human)](http://www.genome.jp/kegg-bin/show_pathway?140722978011892/hsa04978.args) ([5](javascript:display('hsa04978')))

[hsa00052 Galactose metabolism - Homo sapiens (human)](http://www.genome.jp/kegg-bin/show_pathway?140722978011892/hsa00052.args) ([5](javascript:display('hsa00052')))

[hsa00640 Propanoate metabolism - Homo sapiens (human)](http://www.genome.jp/kegg-bin/show_pathway?140722978011892/hsa00640.args) ([5](javascript:display('hsa00640')))

[hsa05340 Primary immunodeficiency - Homo sapiens (human)](http://www.genome.jp/kegg-bin/show_pathway?140722978011892/hsa05340.args) ([5](javascript:display('hsa05340')))

[hsa00514 Other types of O-glycan biosynthesis - Homo sapiens (human)](http://www.genome.jp/kegg-bin/show_pathway?140722978011892/hsa00514.args) ([5](javascript:display('hsa00514')))

[hsa04672 Intestinal immune network for IgA production - Homo sapiens (human)](http://www.genome.jp/kegg-bin/show_pathway?140722978011892/hsa04672.args) ([5](javascript:display('hsa04672')))

[hsa00670 One carbon pool by folate - Homo sapiens (human)](http://www.genome.jp/kegg-bin/show_pathway?140722978011892/hsa00670.args) ([4](javascript:display('hsa00670')))

[hsa05330 Allograft rejection - Homo sapiens (human)](http://www.genome.jp/kegg-bin/show_pathway?140722978011892/hsa05330.args) ([4](javascript:display('hsa05330')))

[hsa05320 Autoimmune thyroid disease - Homo sapiens (human)](http://www.genome.jp/kegg-bin/show_pathway?140722978011892/hsa05320.args) ([4](javascript:display('hsa05320')))

[hsa00062 Fatty acid elongation - Homo sapiens (human)](http://www.genome.jp/kegg-bin/show_pathway?140722978011892/hsa00062.args) ([4](javascript:display('hsa00062')))

[hsa05150 Staphylococcus aureus infection - Homo sapiens (human)](http://www.genome.jp/kegg-bin/show_pathway?140722978011892/hsa05150.args) ([4](javascript:display('hsa05150')))

[hsa00100 Steroid biosynthesis - Homo sapiens (human)](http://www.genome.jp/kegg-bin/show_pathway?140722978011892/hsa00100.args) ([4](javascript:display('hsa00100')))

[hsa04640 Hematopoietic cell lineage - Homo sapiens (human)](http://www.genome.jp/kegg-bin/show_pathway?140722978011892/hsa04640.args) ([4](javascript:display('hsa04640')))

[hsa05144 Malaria - Homo sapiens (human)](http://www.genome.jp/kegg-bin/show_pathway?140722978011892/hsa05144.args) ([4](javascript:display('hsa05144')))

[hsa04610 Complement and coagulation cascades - Homo sapiens (human)](http://www.genome.jp/kegg-bin/show_pathway?140722978011892/hsa04610.args) ([4](javascript:display('hsa04610')))

[hsa00650 Butanoate metabolism - Homo sapiens (human)](http://www.genome.jp/kegg-bin/show_pathway?140722978011892/hsa00650.args) ([4](javascript:display('hsa00650')))

[hsa04744 Phototransduction - Homo sapiens (human)](http://www.genome.jp/kegg-bin/show_pathway?140722978011892/hsa04744.args) ([4](javascript:display('hsa04744')))

[hsa00030 Pentose phosphate pathway - Homo sapiens (human)](http://www.genome.jp/kegg-bin/show_pathway?140722978011892/hsa00030.args) ([4](javascript:display('hsa00030')))

[hsa00380 Tryptophan metabolism - Homo sapiens (human)](http://www.genome.jp/kegg-bin/show_pathway?140722978011892/hsa00380.args) ([4](javascript:display('hsa00380')))

[hsa00512 Mucin type O-Glycan biosynthesis - Homo sapiens (human)](http://www.genome.jp/kegg-bin/show_pathway?140722978011892/hsa00512.args) ([4](javascript:display('hsa00512')))

[hsa05143 African trypanosomiasis - Homo sapiens (human)](http://www.genome.jp/kegg-bin/show_pathway?140722978011892/hsa05143.args) ([4](javascript:display('hsa05143')))

[hsa00051 Fructose and mannose metabolism - Homo sapiens (human)](http://www.genome.jp/kegg-bin/show_pathway?140722978011892/hsa00051.args) ([4](javascript:display('hsa00051')))

[hsa03410 Base excision repair - Homo sapiens (human)](http://www.genome.jp/kegg-bin/show_pathway?140722978011892/hsa03410.args) ([4](javascript:display('hsa03410')))

[hsa00920 Sulfur metabolism - Homo sapiens (human)](http://www.genome.jp/kegg-bin/show_pathway?140722978011892/hsa00920.args) ([4](javascript:display('hsa00920')))

[hsa00410 beta-Alanine metabolism - Homo sapiens (human)](http://www.genome.jp/kegg-bin/show_pathway?140722978011892/hsa00410.args) ([4](javascript:display('hsa00410')))

[hsa00740 Riboflavin metabolism - Homo sapiens (human)](http://www.genome.jp/kegg-bin/show_pathway?140722978011892/hsa00740.args) ([3](javascript:display('hsa00740')))

[hsa00340 Histidine metabolism - Homo sapiens (human)](http://www.genome.jp/kegg-bin/show_pathway?140722978011892/hsa00340.args) ([3](javascript:display('hsa00340')))

[hsa04122 Sulfur relay system - Homo sapiens (human)](http://www.genome.jp/kegg-bin/show_pathway?140722978011892/hsa04122.args) ([3](javascript:display('hsa04122')))

[hsa04964 Proximal tubule bicarbonate reclamation - Homo sapiens (human)](http://www.genome.jp/kegg-bin/show_pathway?140722978011892/hsa04964.args) ([3](javascript:display('hsa04964')))

[hsa00532 Glycosaminoglycan biosynthesis - chondroitin sulfate / dermatan sulfate - Homo sapiens (human)](http://www.genome.jp/kegg-bin/show_pathway?140722978011892/hsa00532.args) ([3](javascript:display('hsa00532')))

[hsa01210 2-Oxocarboxylic acid metabolism - Homo sapiens (human)](http://www.genome.jp/kegg-bin/show_pathway?140722978011892/hsa01210.args) ([3](javascript:display('hsa01210')))

[hsa03450 Non-homologous end-joining - Homo sapiens (human)](http://www.genome.jp/kegg-bin/show_pathway?140722978011892/hsa03450.args) ([3](javascript:display('hsa03450')))

[hsa00040 Pentose and glucuronate interconversions - Homo sapiens (human)](http://www.genome.jp/kegg-bin/show_pathway?140722978011892/hsa00040.args) ([3](javascript:display('hsa00040')))

[hsa04940 Type I diabetes mellitus - Homo sapiens (human)](http://www.genome.jp/kegg-bin/show_pathway?140722978011892/hsa04940.args) ([3](javascript:display('hsa04940')))

[hsa04975 Fat digestion and absorption - Homo sapiens (human)](http://www.genome.jp/kegg-bin/show_pathway?140722978011892/hsa04975.args) ([3](javascript:display('hsa04975')))

[hsa04742 Taste transduction - Homo sapiens (human)](http://www.genome.jp/kegg-bin/show_pathway?140722978011892/hsa04742.args) ([3](javascript:display('hsa04742')))

[hsa00910 Nitrogen metabolism - Homo sapiens (human)](http://www.genome.jp/kegg-bin/show_pathway?140722978011892/hsa00910.args) ([3](javascript:display('hsa00910')))

[hsa00450 Selenocompound metabolism - Homo sapiens (human)](http://www.genome.jp/kegg-bin/show_pathway?140722978011892/hsa00450.args) ([3](javascript:display('hsa00450')))

[hsa00601 Glycosphingolipid biosynthesis - lacto and neolacto series - Homo sapiens (human)](http://www.genome.jp/kegg-bin/show_pathway?140722978011892/hsa00601.args) ([3](javascript:display('hsa00601')))

[hsa02010 ABC transporters - Homo sapiens (human)](http://www.genome.jp/kegg-bin/show_pathway?140722978011892/hsa02010.args) ([3](javascript:display('hsa02010')))

[hsa05332 Graft-versus-host disease - Homo sapiens (human)](http://www.genome.jp/kegg-bin/show_pathway?140722978011892/hsa05332.args) ([3](javascript:display('hsa05332')))

[hsa00565 Ether lipid metabolism - Homo sapiens (human)](http://www.genome.jp/kegg-bin/show_pathway?140722978011892/hsa00565.args) ([3](javascript:display('hsa00565')))

[hsa00531 Glycosaminoglycan degradation - Homo sapiens (human)](http://www.genome.jp/kegg-bin/show_pathway?140722978011892/hsa00531.args) ([3](javascript:display('hsa00531')))

[hsa00053 Ascorbate and aldarate metabolism - Homo sapiens (human)](http://www.genome.jp/kegg-bin/show_pathway?140722978011892/hsa00053.args) ([3](javascript:display('hsa00053')))

[hsa00860 Porphyrin and chlorophyll metabolism - Homo sapiens (human)](http://www.genome.jp/kegg-bin/show_pathway?140722978011892/hsa00860.args) ([3](javascript:display('hsa00860')))

[hsa00590 Arachidonic acid metabolism - Homo sapiens (human)](http://www.genome.jp/kegg-bin/show_pathway?140722978011892/hsa00590.args) ([3](javascript:display('hsa00590')))

[hsa03030 DNA replication - Homo sapiens (human)](http://www.genome.jp/kegg-bin/show_pathway?140722978011892/hsa03030.args) ([3](javascript:display('hsa03030')))

[hsa00770 Pantothenate and CoA biosynthesis - Homo sapiens (human)](http://www.genome.jp/kegg-bin/show_pathway?140722978011892/hsa00770.args) ([3](javascript:display('hsa00770')))

[hsa00563 Glycosylphosphatidylinositol(GPI)-anchor biosynthesis - Homo sapiens (human)](http://www.genome.jp/kegg-bin/show_pathway?140722978011892/hsa00563.args) ([3](javascript:display('hsa00563')))

[hsa00630 Glyoxylate and dicarboxylate metabolism - Homo sapiens (human)](http://www.genome.jp/kegg-bin/show_pathway?140722978011892/hsa00630.args) ([3](javascript:display('hsa00630')))

[hsa03430 Mismatch repair - Homo sapiens (human)](http://www.genome.jp/kegg-bin/show_pathway?140722978011892/hsa03430.args) ([3](javascript:display('hsa03430')))

[hsa00072 Synthesis and degradation of ketone bodies - Homo sapiens (human)](http://www.genome.jp/kegg-bin/show_pathway?140722978011892/hsa00072.args) ([3](javascript:display('hsa00072')))

[hsa00592 alpha-Linolenic acid metabolism - Homo sapiens (human)](http://www.genome.jp/kegg-bin/show_pathway?140722978011892/hsa00592.args) ([2](javascript:display('hsa00592')))

[hsa00360 Phenylalanine metabolism - Homo sapiens (human)](http://www.genome.jp/kegg-bin/show_pathway?140722978011892/hsa00360.args) ([2](javascript:display('hsa00360')))

[hsa00500 Starch and sucrose metabolism - Homo sapiens (human)](http://www.genome.jp/kegg-bin/show_pathway?140722978011892/hsa00500.args) ([2](javascript:display('hsa00500')))

[hsa00533 Glycosaminoglycan biosynthesis - keratan sulfate - Homo sapiens (human)](http://www.genome.jp/kegg-bin/show_pathway?140722978011892/hsa00533.args) ([2](javascript:display('hsa00533')))

[hsa00350 Tyrosine metabolism - Homo sapiens (human)](http://www.genome.jp/kegg-bin/show_pathway?140722978011892/hsa00350.args) ([2](javascript:display('hsa00350')))

[hsa00983 Drug metabolism - other enzymes - Homo sapiens (human)](http://www.genome.jp/kegg-bin/show_pathway?140722978011892/hsa00983.args) ([2](javascript:display('hsa00983')))

[hsa00760 Nicotinate and nicotinamide metabolism - Homo sapiens (human)](http://www.genome.jp/kegg-bin/show_pathway?140722978011892/hsa00760.args) ([2](javascript:display('hsa00760')))

[hsa05310 Asthma - Homo sapiens (human)](http://www.genome.jp/kegg-bin/show_pathway?140722978011892/hsa05310.args) ([2](javascript:display('hsa05310')))

[hsa04614 Renin-angiotensin system - Homo sapiens (human)](http://www.genome.jp/kegg-bin/show_pathway?140722978011892/hsa04614.args) ([1](javascript:display('hsa04614')))

[hsa00400 Phenylalanine, tyrosine and tryptophan biosynthesis - Homo sapiens (human)](http://www.genome.jp/kegg-bin/show_pathway?140722978011892/hsa00400.args) ([1](javascript:display('hsa00400')))

[hsa00830 Retinol metabolism - Homo sapiens (human)](http://www.genome.jp/kegg-bin/show_pathway?140722978011892/hsa00830.args) ([1](javascript:display('hsa00830')))

[hsa00730 Thiamine metabolism - Homo sapiens (human)](http://www.genome.jp/kegg-bin/show_pathway?140722978011892/hsa00730.args) ([1](javascript:display('hsa00730')))

[hsa00524 Butirosin and neomycin biosynthesis - Homo sapiens (human)](http://www.genome.jp/kegg-bin/show_pathway?140722978011892/hsa00524.args) ([1](javascript:display('hsa00524')))

[hsa00140 Steroid hormone biosynthesis - Homo sapiens (human)](http://www.genome.jp/kegg-bin/show_pathway?140722978011892/hsa00140.args) ([1](javascript:display('hsa00140')))

[hsa00750 Vitamin B6 metabolism - Homo sapiens (human)](http://www.genome.jp/kegg-bin/show_pathway?140722978011892/hsa00750.args) ([1](javascript:display('hsa00750')))

[hsa00430 Taurine and hypotaurine metabolism - Homo sapiens (human)](http://www.genome.jp/kegg-bin/show_pathway?140722978011892/hsa00430.args) ([1](javascript:display('hsa00430')))

[hsa00534 Glycosaminoglycan biosynthesis - heparan sulfate / heparin - Homo sapiens (human)](http://www.genome.jp/kegg-bin/show_pathway?140722978011892/hsa00534.args) ([1](javascript:display('hsa00534')))

[hsa05033 Nicotine addiction - Homo sapiens (human)](http://www.genome.jp/kegg-bin/show_pathway?140722978011892/hsa05033.args) ([1](javascript:display('hsa05033')))

[hsa00604 Glycosphingolipid biosynthesis - ganglio series - Homo sapiens (human)](http://www.genome.jp/kegg-bin/show_pathway?140722978011892/hsa00604.args) ([1](javascript:display('hsa00604')))

[hsa00120 Primary bile acid biosynthesis - Homo sapiens (human)](http://www.genome.jp/kegg-bin/show_pathway?140722978011892/hsa00120.args) ([1](javascript:display('hsa00120')))

[hsa04977 Vitamin digestion and absorption - Homo sapiens (human)](http://www.genome.jp/kegg-bin/show_pathway?140722978011892/hsa04977.args) ([1](javascript:display('hsa04977')))

[hsa00471 D-Glutamine and D-glutamate metabolism - Homo sapiens (human)](http://www.genome.jp/kegg-bin/show_pathway?140722978011892/hsa00471.args) ([1](javascript:display('hsa00471')))
